# Biopolymer-based electrospun nanofiber membranes for smart food packaging applications: a review

**DOI:** 10.1039/d5ra02348c

**Published:** 2025-06-25

**Authors:** Maria Mathew, Sagitha Paroly, Sujith Athiyanathil

**Affiliations:** a Material Research Laboratory, Department of Chemistry, National Institute of Technology Calicut Kerala 673601 India sujith@nitc.ac.in athiyanathil.sujith@gmail.com

## Abstract

Food safety is an important concern impacting public health and the well-being of society. Novel food packaging systems are adopted by the market owing to the increased consumer preference for healthy food products. To minimize food spoilage, it is essential to monitor the food quality throughout the food supply chain in real time. The development of food packaging systems such as active and intelligent packaging has been greatly accelerated to meet these requirements. It relies on the development of new materials and technologies to monitor the quality, shelf life, and usability of food products for its end-users. Polymeric nanofibers, which can be easily functionalized, are good candidates for these applications. This review describes the role of electrospun nanofibers in fabricating various active and intelligent food packaging systems. Electrospun nanofibers encapsulated with bioactive ingredients can serve as a promising and environmentally sustainable alternative to conventional plastic systems for widespread use. Moreover, nanofiber packaging materials have many functional advantages such as controlled release of active agents, gas permeability, and flexibility. This review will be a valuable source of information for a wide group of researchers working in the area of electrospinning and biopolymer-based active and intelligent food packaging systems.

## Abstract 2

Encapsulation of bioactive agents in nanofiber membranes for smart food packaging application.

## Graphical abstract

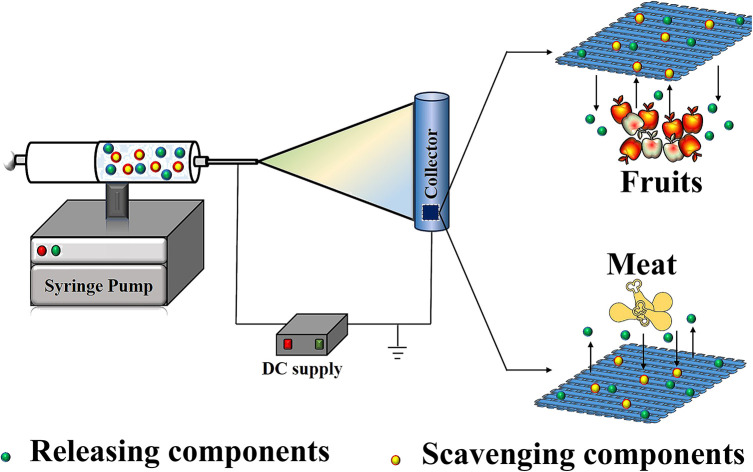

## 1. Introduction

Food packaging is considered a crucial step in the food production process as it has a significant impact on the quality, safety, and freshness of food items. It acts as a barrier to various contaminants, including air, light, moisture, microbes, and many physical and chemical pollutants.^1^ Traditional food packaging is meant to only provide physical support and protection against external environmental stimuli such as light, temperature, moisture, oxidation, and various microbial contaminations.^2^ This packaging ensures the primary purpose of protecting a product during the processes of transportation, distribution, and storage.^3^ Materials that have traditionally been used for food packaging include glass, metals (aluminium foils and laminates, tin plates, and steel), paper, and plastics. However, food deterioration during distribution, transport, and storage results in food loss and food-related health issues. Proper food packaging can reduce these issues to a great extent. Considering the increased demand for improving the shelf life of food products and real-time quality monitoring, traditional food packaging is being replaced by smart packaging technologies. Smart food packaging is an innovative packaging solution that can detect, record, and sense the quality of food products and convey efficiently to the consumers. Smart food packaging utilizes sensors or indicators that can detect and analyse various factors such as freshness, pathogens, pH, CO_2,_ oxygen, or temperature.^4^

Active food packaging and intelligent food packaging are the two broad classifications of smart “interactive” food packaging. Active food packaging involves the incorporation of certain active components into the packaging material that can release antioxidants, antibacterial agents, CO_2_ emitters, *etc.* Additionally, they can absorb moisture, ethylene gas, and oxygen from the packaging itself and its surrounding environment.^5^ Thus, an active packaging system modifies the packaging environment during the preservation period without compromising the quality and safety of packaged foods. The next category of smart food packaging system is intelligent packaging. The goal of intelligent food packaging is to convey information regarding food freshness *via* various signals, *i.e.* using sensors, indicators, and data carriers.^6^ Recently, such smart food packaging has attracted great attention due to its ability for the real-time monitoring of food freshness and spoilage. It also enables the tracking and tracing of a product throughout its lifecycle, and this information can be utilized by the manufacturers, retailers, or consumers to understand the real-time condition of the products.^7^

To fulfill the criteria for smart food packaging, the active agents integrated within the packaging should exhibit exceptional stability, enabling their controlled and gradual release without any loss or degradation. Among the various techniques employed in food packaging, membrane-based processes are particularly favored due to their adaptability, cost-effectiveness, low energy requirements, high reliability, ease of scale-up, and environmentally sustainable nature.^8^ Several methods such as solution casting, 3D printing, sol–gel method, extrusion, and electrospinning are available for the fabrication of smart food packaging.^9^ However, since most active agents evaporate easily due to their high volatility, it is not feasible to incorporate them directly through conventional polymer processing methods. To overcome these problems, electrospinning technique can be employed due to its simplicity and cost-effectiveness in producing nanofibers with large surface area, porosity, flexibility, and other tunable features. Nanofibers are typically defined as fibers having diameters less than 1000 nm.^10^ Due to the high encapsulation efficiency, electrospun nanofibers can act as excellent carriers of various active agents for smart food packaging applications.^11,12^ Electrospinning offers several advantages over the conventional encapsulation and film-forming methods. It enables the production of nanofiber membranes with a high surface area-to-volume ratio and tunable porosity, enhancing barrier properties and functional activities. The process allows for the efficient encapsulation of bioactive agents (antioxidants, antibacterial agents, enzymes, and prebiotics) under mild conditions. The introduction of active agents into electrospun membranes can be achieved through two main approaches. The first method involves the incorporation of agents directly into the electrospinning solution, *via* dissolving or dispersing the agents within the polymer matrix.^13^ The second approach relies on post-treatment methods such as grafting, dip coating, and surface adsorption.^14^ Incorporation during electrospinning ensures uniform distribution and better encapsulation, while post-treatment allows for surface functionalization without altering the fiber structure. This is particularly advantageous for preserving the structural integrity and functionality of heat-sensitive compounds such as essential oils, antioxidants, and antimicrobials. In contrast, conventional encapsulation techniques require elevated temperatures or harsh chemical conditions, which can badly affect the functional properties of bioactive compounds. Moreover, electrospinning is a relatively simple, portable, and low-cost technique that requires minimal manpower, making it suitable for both laboratory and industrial settings. Its versatility in polymer selection and precise control over fiber morphology, mechanical, and transport properties further support its application in developing advanced, sustainable food packaging materials. Additionally, over the years, electrospinning has been developed with diverse structures and morphologies to fulfill specific requirements such as uniaxial, core–shell, hollow, and porous structures.^10,15^

A wide range of polymers can be processed through electrospinning to produce fibrous membranes. The use of biopolymers to develop active and intelligent packaging materials can provide excellent biodegradability, biocompatibility, and low toxicity.^16^ To reduce environmental impacts and dependence on fossil fuels, many efforts have been made to substitute synthetic polymers with biodegradable polymers, especially those derived from natural sources. Biopolymers for food packaging can be either extracted directly from biomass or produced by microorganisms or synthesized from bioderived monomers.^17,18^

The existing research studies have independently explored various aspects of electrospinning and smart food packaging.^19,20^ Our review presents a comprehensive and integrative analysis of biopolymeric electrospun membranes within the context of smart food packaging, setting it apart from the existing literature. We investigate the principles of both active and intelligent packaging systems, elucidating their functionalities, and the synergistic role of electrospun biopolymeric membranes in enhancing these systems. A detailed examination of the electrospinning process, including critical factors influencing the fiber morphology and functionality, which is essential for tailoring packaging materials to specific food preservation applications is also provided in this review. We emphasize the use of sustainable and biodegradable biopolymers in electrospinning, aligning with environmental concerns for developing eco-friendly packaging solutions. Furthermore, we highlight the capacity of electrospun membranes to encapsulate bioactive agents to facilitate their sustained release, a feature pivotal for extending the shelf life and ensuring food safety. By critically assessing recent studies, identifying gaps, and proposing future research outlook, our review serves as a consolidated resource that not only summarizes current advancements but also provides strategic insights for future research and development in smart food packaging technologies.

## 2. Active packaging

**Fig. 1:**
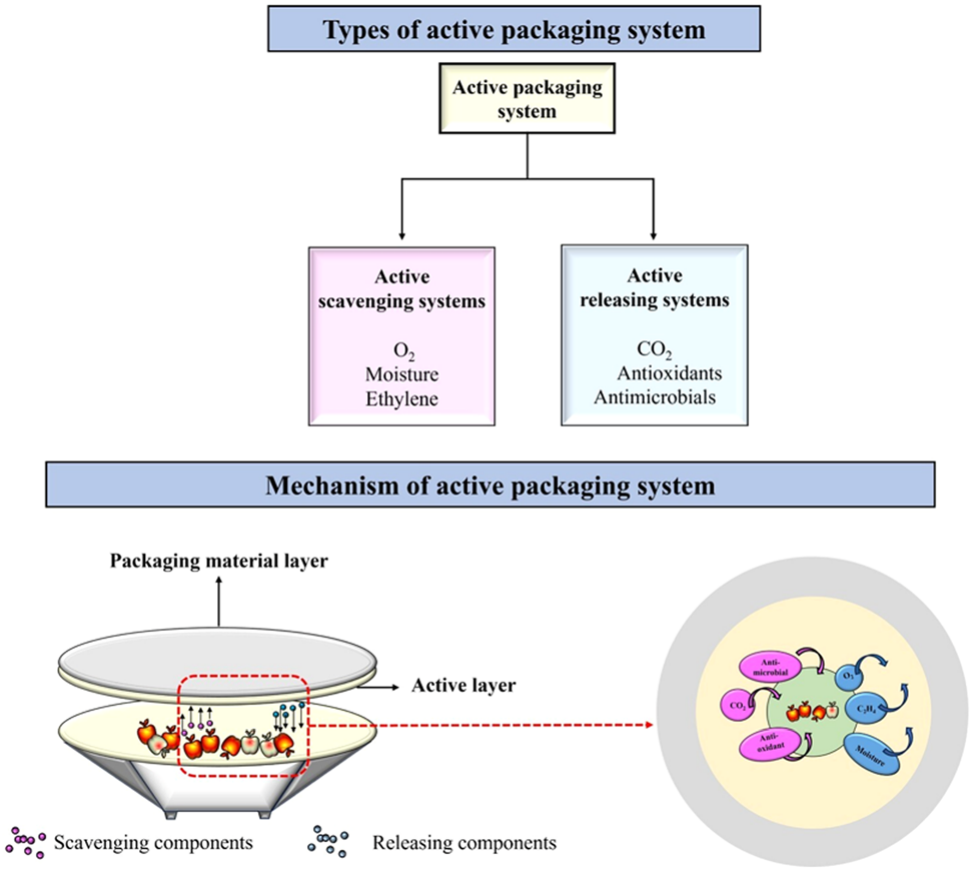
Schematics representing the basics of active packaging systems.

Active food packaging is meant to improve the properties of packaged food products by maintaining their quality and extending their shelf life. According to European regulation No. (EC) 450/2009, active food packaging systems are designed to “*deliberately incorporate components that would release or absorb substances into or from the packaged food or the environment surrounding the food”*.^21^ The active components are generally incorporated into the bulk of the food. However, for the majority of fresh and processed foods, food degradation and microbial growth occur on the surfaces of food. Moreover, certain active components when added directly to food will undergo unwanted chemical interactions with the food components causing the inhibition or reduction of their activity. Therefore, adding active components to the packaging system is the most efficient way compared to adding them directly to the food. The active packaging systems are classified into two types: active scavenging systems and active releasing systems, as shown in Fig. 1. The active scavenging systems are also known as ‘absorbers’, which can absorb components such as ethylene, oxygen, and moisture. This category includes ethylene scavengers, moisture scavengers, and oxygen scavengers. In contrast, the active releasing systems are known as ‘emitters’, which can emit active components such as antioxidants, antimicrobials and carbon dioxide. Hence, the active releasing systems are classified as antioxidant emitters, antimicrobial emitters, and carbon dioxide emitters.^22^

### 2.1. Ethylene scavengers

Ethylene (C_2_H_4_) is one of the most important plant hormones released from fruits and vegetables, which controls physiological mechanisms, growth, ripening, and senescence. Ethylene also stimulates chlorophyll loss and enhances excessive softening of climacteric fruits such as apple, tomato, avocado, banana, pear, mango, and kiwi.^23^ Mainly, there are two types of ethylene responsible for the various physiological changes in fruits. It includes endogenous ethylene, produced biologically by plants, and exogenous ethylene generated by external sources such as neighbouring crops, automotive exhaust, and tobacco.^24^ Exogenous ethylene is responsible for the browning and peel color transformation in postharvest fruits. Since most fruits and vegetables are ethylene sensitive, prolonged exposure to ethylene often leads to over-ripening, spoilage, change in taste, color, and odor. Therefore, ethylene scavengers are used in food packages to limit ethylene accumulation for retaining the quality and thereby extending the shelf life of fresh produce.^25^ Ethylene scavengers will absorb ethylene gas produced during storage either *via* physical or *via* chemical adsorption processes. The ethylene scavengers include inhibitors, adsorbents, and catalytic oxidants. Specific examples of each category are summarized in Table 1.

**Table 1:** Different categories and respective examples of ethylene scavengers for food packaging applications

Type of ethylene scavengers	Examples
Inhibitors	1-Methylcyclopropene (1-MCP), silver (Ag) ions, 2-aminoethoxyvinyl glycine (AVG)
Adsorbents	Natural clays like montmorillonite, halloysite nanotubes, activated charcoal, aluminosilicates, silica gel, palladium-based systems
Catalytic oxidants	Potassium permanganate (KMnO_4_), ozone (O_3_), titanium dioxide (TiO_2_)

Potassium permanganate (KMnO_4_)-based ethylene scavengers are the most studied systems. KMnO_4_ functions as a potent oxidizing agent, facilitating the removal of ethylene (C_2_H_4_) *via* an oxidation reaction. At the molecular level, KMnO_4_ oxidizes ethylene by cleaving its double bond, subsequently converting it into carbon dioxide (CO_2_) and water (H_2_O), while KMnO_4_ itself undergoes reduction to form manganese dioxide (MnO_2_). The simplified reaction is represented in eqn (1):^26^

1 3C_2_H_4_ + 12KMnO_4_ + 18H_2_O → 12MnO_2_ + 12KOH + 6CO_2_

This redox reaction is characterized by a visible color transition from purple (KMnO_4_) to brown (MnO_2_), which serves as an indicator of the scavenger's saturation and subsequent loss of activity. The oxidation process occurs on the surface of the support material, typically initiated by the physisorption of ethylene, followed by chemisorption and reaction with KMnO_4_.

**Fig. 2:**
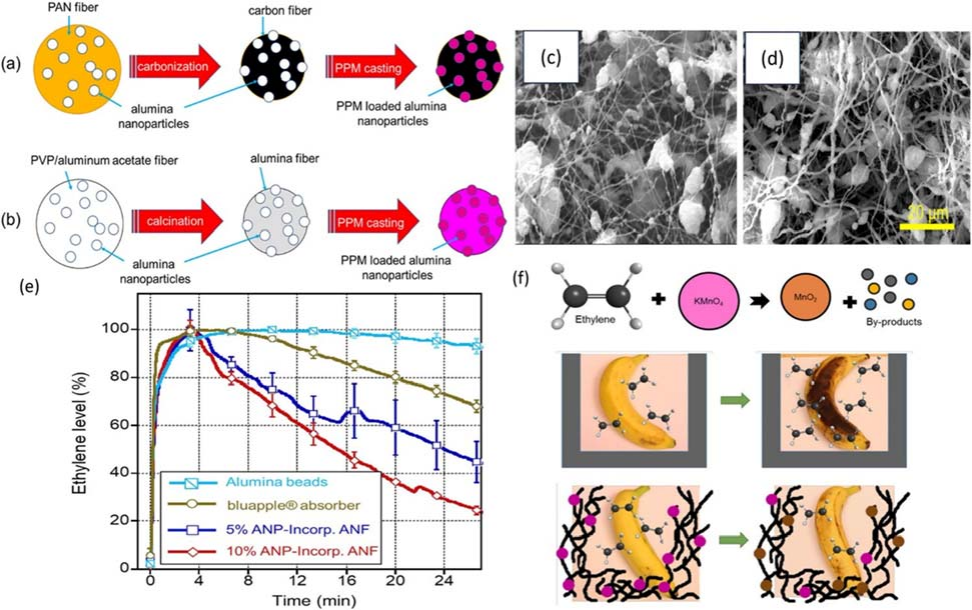
Production of ANP-incorporated (a) CNFs and (b) ANFs followed by PPM casting. SEM images of ANP-incorporated (c) carbon nanofibers and (d) alumina nanofibers. (e) Ethylene absorption capacity for various absorbers. (f) Electrospun nanofiber membranes as ethylene scavengers.^27^. [Copyright © 2018, American Chemical Society].

However, due to the high sensitivity of KMnO_4_, it can be deactivated during membrane or film processing. Consequently, Tirgar *et al.*^27^ proposed an innovative method for incorporating potassium permanganate (PPM) into electrospun nanofiber membranes to delay banana ripening. This study employs two distinct approaches for fabricating PPM-loaded nanofibers. The first approach utilizes carbon nanofibers (CNFs) loaded with alumina nanoparticles (ANPs) as carriers for KMnO_4_ (Fig. 2a), as carbonized fibers are stable and non-reactive with KMnO_4_. The second approach involves alumina nanofibers (ANFs) loaded with ANPs as a support for PPM, offering high surface area and stability (Fig. 2b). When comparing the membrane absorption capacity with various forms of ethylene scavengers such as beads, films, and liners, the alumina nanofiber membrane, with its exceptionally high surface area, demonstrated a superior ethylene absorption capacity over an extended period. However, the main disadvantage of KMnO_4_-based systems is that they cannot be used in those packages that make direct contact with food because of their toxicity. Hence, generally safe and non-toxic adsorbents incorporated in polymeric films are always receiving popularity. The main non-toxic adsorbents used for ethylene removal include zeolites, clay, alumina, silica, and activated carbon. The high specific surface area of these porous materials makes them capable of absorbing ethylene released from fruits and vegetables effectively. These materials exhibit distinct adsorption isotherms based on their surface chemistry, pore structure, and interaction mechanisms with ethylene. Silica typically exhibits physical adsorption, due to its relatively low surface energy and mesoporous structure.^28^ Zeolites, being microporous and highly crystalline with well-defined pore sizes and surface polarities, often follow Langmuir-type isotherms, indicating monolayer chemisorption or selective adsorption based on molecular sieving.^29^ In contrast, activated carbon, with its high surface area and heterogeneous pore structure, supports both physisorption and chemisorption, depending on surface functional groups and activation conditions. Due to this variation in isotherm behavior, it is important to select appropriate scavengers based on the desired adsorption mechanism and application.

The chitosan/zeolite packaging film developed by Sousa *et al.*,^30^ the corn starch-gum acacia film impregnated with sepiolite clay reported by Upadhyay *et al.*,^31^ the dendritic mesoporous silica-supported platinum catalyst as an ethylene scavenger developed by Wei *et al.*^32^ and the palladium-modified activated carbon-based ethylene adsorbent for delaying the ripening of banana^33^ are few examples of recent research works on ethylene scavengers. Recently, a group of researchers have reported that brick ash, the industrial byproduct of brick kilns can exhibit excellent ethylene adsorption capacity at room temperature. Suraj *et al.*^34^ activated the brick ash at a higher temperature to study its adsorption properties, and it was observed that the material showed higher ethylene adsorption capacity as the activation temperature varied from 500 °C to 1000 °C. The practical applicability of this newly developed material was evaluated through shelf-life studies on raw bananas at 22–24 °C temperature and 65% relative humidity (RH) conditions. The results indicated that the new material could provide an increased shelf life of up to 20 days, compared to the control (12 days).

**Fig. 3:**
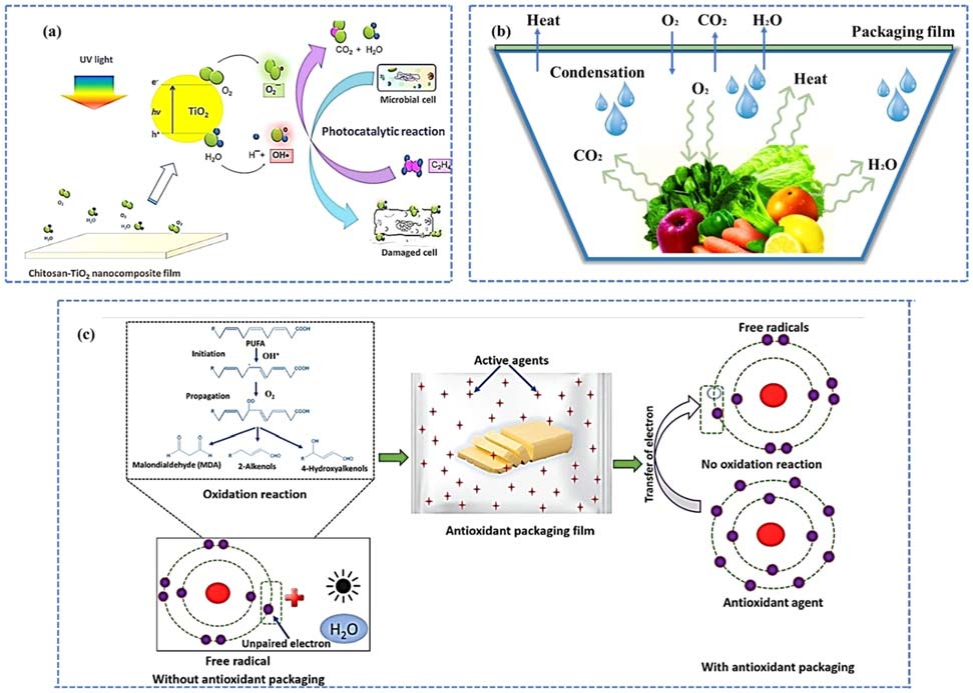
Schematics: (a) mechanism of photocatalytic degradation of ethylene and antimicrobial activity of the chitosan-TiO_2_ film [reprinted with permission from ref. 37 Copyright Elsevier 2018]. (b) Moisture condensation mechanism in packaging trays containing fresh produces. (c) Oxidation mechanism (i) without and (ii) with antioxidants in the packaging material [reproduced with permission from ref. 38 Springer Nature].

Photocatalytic oxidation is another effective way of ethylene removal for the preservation of postharvest fruits and vegetables. This technique involves the use of photocatalysts such as TiO_2_, which get activated upon irradiation with UV light and facilitate the oxidation of ethylene to CO_2_ and H_2_O.^35,36^Fig. 3a shows the schematic diagram representing the mechanism of photocatalytic degradation of ethylene and the antimicrobial activity of the chitosan-TiO_2_ film developed by Siripatrawan *et al.*^37^ Reactive oxygen species (ROS) such as hydroxyl radicals (OH) and superoxide ions (O_2_^2−^) generated upon UV irradiation react with ethylene and polyunsaturated phospholipids in the cell membrane of microorganisms. Even though this technique has been extensively studied for air and water purification, only limited attention has been paid to the postharvest preservation of fruits and vegetables.

### 2.2. Moisture scavengers

Moisture content and elevated relative humidity (RH) are critical factors influencing the quality, safety, and shelf life of food products. Excess moisture accelerates microbial spoilage, promotes lipid oxidation, and induces undesirable changes in texture and appearance, thereby significantly reducing product stability and consumer acceptability. Fish, meat, poultry, and other minimally processed fruits and vegetables are examples of highly moisture-sensitive food items. For these types of water-sensitive products, controlled moisture levels are essential for keeping them safe from microbial growth to enhance their shelf life and sensory properties. Moisture accumulation within packaged food products can occur as a result of excessive transpiration and condensation (Fig. 3b). Both transpiration and condensation are key factors that can negatively impact postharvest quality by causing defects in external appearance and promoting the growth of spoilage microorganisms.^39,40^ Therefore, achieving a stable temperature-humidity equilibrium within the packaging system is essential for extending the shelf life of fresh produce and maintaining product integrity.^41^ Transpiration is a physiological process through which moisture moves from the internal tissues of the product to its surface and eventually into the surrounding air. This movement is driven by differences in moisture levels between the product and its environment, specifically differences in water activity, moisture concentration gradient, and water vapor pressure. However, under saturated storage conditions (100% relative humidity and constant temperature), transpiration can still occur, primarily due to the internal heat generated by the respiration process. When the water vapor transmission rate (WVTR) of the packaging material is lower than the product's transpiration rate, the moisture released by the product cannot escape efficiently. Consequently, water vapor accumulates inside the package, causing an increase in internal water vapor pressure, eventually leading to condensation on the inner surface. Similarly, once the interior surface temperature of the packaging falls below the dew point of the enclosed air, condensation occurs. This results in the formation of liquid droplets on the internal surfaces. This process is driven by vapor pressure differentials between the food product, the package headspace, and the external environment. Temperature fluctuations during storage and transportation further accelerate these differentials, promoting internal moisture migration and droplet formation.

Similarly, the action of metabolizing fats, carbohydrates, and other biological compounds can also generate water. As a result, food with a higher moisture content tends to exhibit elevated vapor pressure, and the degradation of these organic molecules results in the accumulation of moisture within the headspace of food packages. An effective way to control this excess moisture is to use an appropriate moisture absorber/scavenger.^42^ Moisture absorbers or scavengers are usually incorporated inside sachets, pads, and films to maintain relative humidity (RH) within packages. Desiccants are examples of materials used to remove moisture in packaging headspace. Desiccants work by absorbing or adsorbing water molecules, thereby reducing humidity and moisture-activated microbial growth and food spoilage.^43^ Conventional drying methods require high-temperature conditions.^44,45^ However, desiccants are effective for food packaging, as they produce dry air by adsorbing moisture, enabling low-temperature, low-humidity drying ideal for preserving color, texture, and nutrients in heat-sensitive foods. The process relies on vapor pressure differences between the air and the desiccant, and dry desiccants absorb moisture until equilibrium is reached. Common examples of desiccants include silica gel, activated charcoal, activated clay, molecular sieves, and humectant salts (CaCl_2_, MgCl_2_, and CaSO_4_). Generally, desiccants are considered as renewable. To regenerate the desiccant, it must be heated, so its surface vapor pressure exceeds that of the surrounding air. This can be achieved using either renewable (*e.g.*, solar, waste heat) or non-renewable energy sources. New formulations of desiccants allow regeneration at lower temperatures, enhancing the energy efficiency and sustainability. Renewable desiccants, often derived from natural materials such as clay or plant-based substances, are generally more biodegradable and environmentally friendly, but may have lower moisture absorption capacity than synthetic ones. Synthetic desiccants manufactured from chemical processes typically offer higher efficiency, but may have a larger environmental footprint. End-of-life management options for both types include reusability *via* regeneration processes.^41^ Synthetic desiccants (molecular sieves, activated alumina, and phosphorous pentoxide) typically offer more regeneration cycles and can be thermally regenerated multiple times. Biodegradability also varies, with renewable desiccants typically degrading more readily. Certain desiccants can be either recycled or thermally regenerated; however, the associated energy consumption must be carefully evaluated in sustainability assessments. Kenyo *et al.*^46^ studied the effect of desiccant characteristics on the properties of polystyrene/zeolite-active packaging materials using five different categories of zeolites as adsorbents. The results proved that the moisture adsorption capacity is determined by the zeolite content and not its type. In addition to this, super absorbent polymers (SAP) are used as desiccants.

**Fig. 4:**
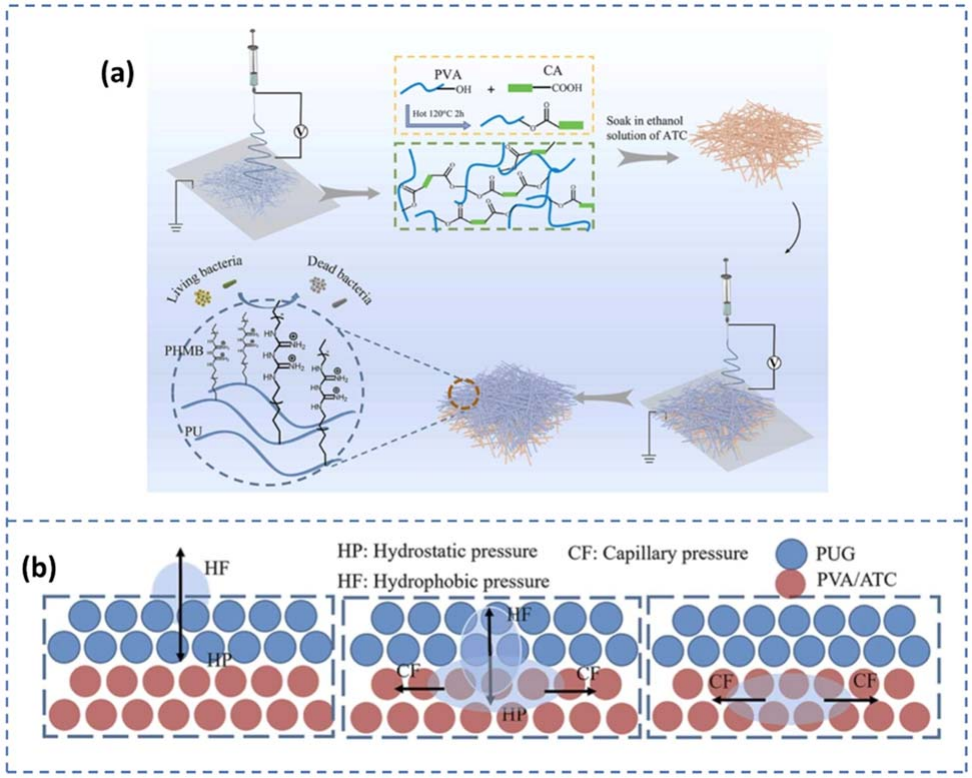
(a) Schematic representation showing the fabrication of antibacterial smart adsorbent pad. (b) Principle behind water conductivity. [Reprinted with permission from ref. 50 Copyright Elsevier 2023].

Superabsorbent polymers (SAPs) are highly hydrophilic materials characterized by a cross-linked network structure, typically in the form of microbeads capable of absorbing and retaining large quantities of water, 1000 times as much as their own weight, significantly exceeding the capacity of conventional hydrogels. Their remarkable absorption capacity is attributed to the presence of strongly hydrophilic functional groups (*e.g.* –OH, –COOH/–COO^−^M^+^, –CONH_2_, and –SO_3_H/–SO_3_^−^M^+^). These groups will undergo strong interactions with water *via* hydrogen bonding and ion-dipole interactions. However, to prevent dissolution in water, a stable three-dimensional cross-linked structure is required, ensuring that the SAPs undergo extensive swelling without dissolving.^47^ Superabsorbent hydrogel films are widely used in active food packaging applications to extend the shelf life of perishable food products.^48^ Superabsorbent polymers (SAPs) can be categorized into natural and synthetic types based on their origin.^49^ Natural polymer-based SAPs include materials such as starch, cellulose, and chitin. These natural SAPs are highly biodegradable, but have a limited capacity for water absorption. In contrast, SAPs derived from synthetic polymers such as polyacrylic acid (PAA), polyacrylamide (PAM), and polyvinyl alcohol (PVA) generally exhibit water absorption and retention capabilities superior to their natural counterparts. However, the degradation of synthetic SAPs in soil takes many years, leading to significant soil pollution. Jiao *et al.*^50^ developed an antibacterial smart adsorbent pad with a Janus structure for preserving meat using the electrospinning technique (Fig. 4a). This pad is composed of two layers, each with distinct wettability. The top layer is made of hydrophobic polyurethane (PU) nanofibers infused with a cationic antimicrobial agent, while the bottom layer is a superabsorbent hydrophilic PVA containing anthocyanins. This dual-layer membrane with varying wettability facilitates the movement of meat juice from the hydrophobic to the hydrophilic layer, with the hydrophobic top layer remaining dry to inhibit bacterial growth. Water conductivity tests demonstrated that liquid moves more swiftly from the hydrophobic side to the hydrophilic side than in the reverse direction. When the hydrophobic force (HF) is less than the hydrophilic pressure (HP), liquid droplets penetrate the hydrophobic layer to reach the hydrophilic layer. The droplets are then subjected to capillary force (CF) from the hydrophilic PVA nanofibers, and quickly spread within the absorbent layer (Fig. 4b). This multifunctional antimicrobial water-absorbing indicator pad offers new insights and methods for meat preservation and freshness indication.

Other approaches for moisture regulation include modified atmospheric packaging (MAP). This kind of packaging can be achieved by exchanging the humid air within the headspace with a dry modified atmosphere gas, usually nitrogen and carbon dioxide. Alternatively, vacuum packaging can be used, where humid air is removed from the headspace.^51^ MAP is particularly effective for perishable foods such as meat, dairy, and fresh produce, as it helps retain freshness, color, texture, and nutritional value without the need for chemical preservatives.

Recently, Kumar *et al.*^52^ have conducted experimental studies on the moisture barrier properties of four different polymeric films including ethylene vinyl alcohol (EVOH), polypropylene (PP), low-density polyethylene (LDPE) and high-density polyethylene (HDPE). The analysis was performed by measuring the water vapor transmission rates under accelerated conditions according to the ASTM standard E96, at 80% relative humidity and 40 °C temperature conditions. The moisture absorption increases linearly with time and becomes constant beyond a specific threshold. Notably, the polypropylene film displayed superior moisture barrier characteristics coupled with robust mechanical and structural stability, making it a cost-effective choice.

### 2.3. Oxygen scavengers

Oxygen scavengers (antioxidants) are additives capable of retarding oxidation reactions that cause rancidity.^53^ Lipid peroxidation is a biochemical process wherein oxidants such as free radicals or nonradical species target lipids, particularly polyunsaturated fatty acids (PUFAs). This involves hydrogen abstraction from a carbon, forming lipid peroxy radicals and hydroperoxides *via* oxygen insertion.^54^ The PUFA oxidation products such as lipid hydroperoxides and secondary reactive aldehydes (malondialdehyde (MDA) and 4-hydroxynonenal (4-HNE)) can disturb cellular homeostasis. These reactive species can create adducts with proteins, lipids, and DNA, resulting in structural damage and functional degradation. For example, 4-HNE functions as a molecular signal that activates genes associated with inflammation, hence contributing to chronic inflammatory states and potentially to diseases such as cancer, atherosclerosis, or neurological disorders.^55,56^ Additionally, lipid peroxides can impair membrane integrity and mitochondrial function, hence intensifying cellular stress.

Another issue related to PUFA-rich food is the off-flavour due to aldehydes, ketones, and volatile secondary oxidation products. For example, 2-pentyl furan and its isomers are responsible for the undesirable “reversion flavor” of soybean oil produced by light-induced singlet oxygen oxidation of linoleic acid.^57^ Thus, in antioxidant packages, oxygen scavengers or oxygen emitters are one of the main components used for preventing uncontrolled radical generation and propagation. Both natural and synthetic antioxidants have been widely used in food packaging to prevent lipid oxidation.^58^Fig. 3c represents the antioxidant mechanism of packaging material.^38^ Natural antioxidants such as tocopherols, flavonoids, and essential oils derived from plant or animal sources are generally regarded as safe. They are attractive due to their biodegradability, lower toxicity, and alignment with consumer trends toward natural ingredients. However, their efficacy may vary based on the source, stability under processing conditions, and extraction costs. Additionally, natural antioxidants often have a lower activity than that of their synthetic counterparts and may impart unwanted flavors or colors to food products. Synthetic antioxidants including butylated hydroxytoluene (BHT), butylated hydroxyanisole (BHA), and *tert*-butylhydroquinone (TBHQ) are chemically synthesized compounds that offer high stability, consistent performance, and cost-effectiveness. They are widely used in the food industry due to their potent antioxidant activity and well-established regulatory frameworks. However, some synthetic antioxidants have raised safety concerns, leading to restrictions or bans in certain countries, and increasing consumer resistance to their use. Therefore, while natural antioxidants are preferable for sustainable and consumer-friendly packaging solutions, synthetic antioxidants continue to play a role where the performance, cost, and regulatory approvals align with application requirements. A hybrid approach or the use of encapsulated natural antioxidants might offer an optimal balance in active food packaging systems.

Vidal *et al.*^59^ developed an antioxidant packaging film by incorporating green coffee oil by-products into a carboxymethyl cellulose film to prevent fish oil oxidation. The capacity of the active film was monitored by analysing peroxides and thiobarbituric acid reactive species (TBARS), primary and secondary products of oxidation, *etc.* Almonds are susceptible to lipid oxidation due to their high content of PUFA, mainly linoleic acid. Almond skin is a potent natural antioxidant that is being incorporated into poly(ε-caprolactone) (PCL) by Garcia *et al.*^60^ to develop an efficient antioxidant food packaging film. Even though no significant differences were observed for the oxygen barrier properties, an increase in water absorption and water vapor permeability was observed for active films compared to the PCL control. This indicates the suitability of this novel packaging for extending the shelf life of fatty foods.

### 2.4. Antimicrobial food packaging systems

Antimicrobial food packaging systems are meant to inhibit the growth of undesired microorganisms responsible for the spoilage of packaged food products. It is an efficient packaging technology, where antimicrobial agents are incorporated into polymeric films that suppress the activities of food-borne pathogens or other targeted microorganisms dangerous to consumer health.^61,62^ Antimicrobial agents can be either directly integrated into food or incorporated into packaging materials, where it is slowly released to kill the pathogens and, thus, maintain the food quality and extend the shelf life. The selection of antimicrobial agents depends on the characteristics of microorganisms. The most commonly used antimicrobial agents for food packaging include essential oils, natural extracts, enzymes, bacteriocins, organic acids, and other derivatives. Close or direct contact with the microorganisms is the most essential requirement for the action of antimicrobial agents. Antimicrobial agents can prevent microbial growth by several mechanisms such as^63^ (i) modifying or denaturing the protein composition within microorganisms, influencing their functionality; (ii) disrupting cell membrane proteins or membrane lipids; (iii) inhibiting the synthesis of cell wall constituents, thus obstructing a stable cell wall structure; and (iv) preventing the reproduction, transcription, and translation of nucleic acids within microorganisms, hindering their ability to replicate. Table 2 summarizes some of the recent research works on antimicrobial food packaging systems.

**Table 2:** Outline of some of the recent research works on antimicrobial food packaging systems^a^

Antimicrobial agents		Target microorganism	Food	Packaging material	References
Essential oils	Thyme–clove EO	*S. aureus*	—	PLA-PBAT	64
		*E. coli*			
	Basil EO	Gram-positive bacteria	Cooked ham	Chitosan	65
	Clove EO	*E. coli*	—	Pullulan/gelatin	66
		*S. aureus*			
	Thyme–cinnamon–oregano EO	*E. coli*	Strawberry	Zein/chitosan	67
		*S. aureus*			
		*C. parapsilosis*			
		*A. brasiliensis*			
	Chrysanthemum EO	*L. monocytogenes*	Beef	Chitosan nanofiber	68
Enzymes	Lysozyme	Gram-positive bacteria	Cheese	_—	69
	Lactoperoxidase	Gram-positive bacteria	Mango	Chitosan	70
		Gram-negative bacteria			
Bacteriocins	Nisin	*M. luteus* ATCC 10240 bacterial flora	Tryptone soya broth (TSB) milk	LDPE	71
	Nisin and EDTA	*E. coli*	Fish fillet	Chitosan-polylactate	72
		*S. aureus*			
Natural extracts	Grape pomace	*E. coli*	—	Polypropylene	73
		*B. subtilis*			
	Date palm fruit	*S. aureus*	Strawberry	Chitosan-PEG	74
	Curcumin	*S. aureus*	Meat	Cellulose laurate	75
Organic acids	Benzoic acid	*E. coli*	Meat	Chitosan	76
		*S. aureus*			
	Sorbic acid	*B. cereus*			
		*P. fluorescens*			
	Acetic acid	*Salmonella*	Meat	—	77
	Citric acid		Poultry products		
	Lactic acid				

a EO: essential oil; PLA: polylactic acid; PBAT: poly(butylene adipate-*co*-terephthalate); LDPE: low-density polyethylene; PEG: polyethylene glycol; *E. coli*: *Escherichia coli*; *S. aureus*: *Staphylococcus aureus*; *C. parapsilosis*: *Candida parapsilosis*; A. brasil.

## 3. Intelligent food packaging

**Fig. 5:**
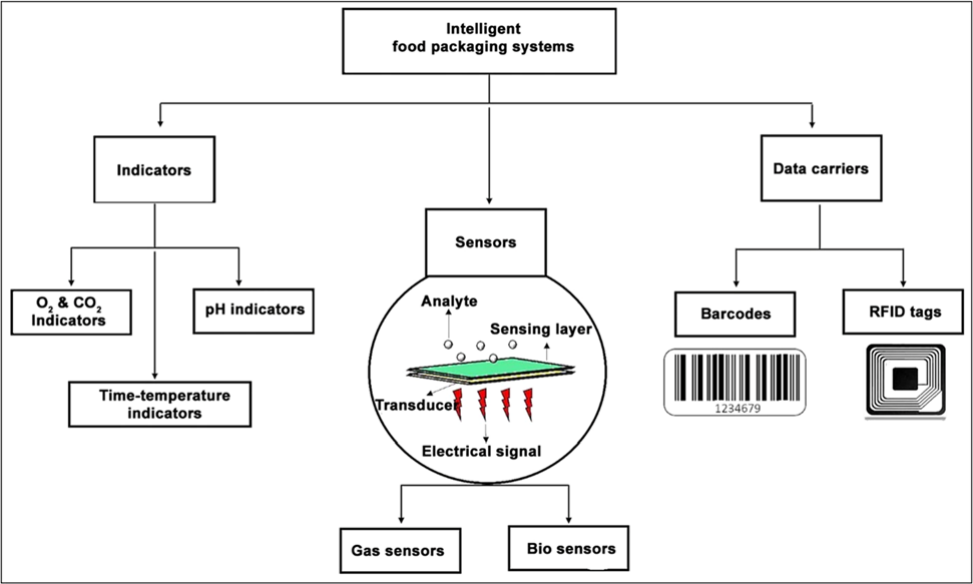
Schematics showing various categories of intelligent food packaging systems.

Even though food quality tests are regularly performed during the production and packing stage, no quality assessments are usually done aftermarket delivery. Therefore, food spoilage during this period could result in significant food loss and consumer health issues. Intelligent food packaging is a smart solution for this scenario. Intelligent packaging is an emerging food packaging technology that is capable of monitoring the condition of packed foods.^78^ It helps to sense and share the quality status of the packed foods with the stake holders of the food supply chain. This technology also helps to monitor the presence of specific chemicals, growth of microbial metabolites, humidity, pH, and gaseous (O_2_ and CO_2_) levels inside the packaging. In general, intelligent food packaging systems rely on three main technologies: indicators, sensors and data carriers (Fig. 5).^79^

In smart food packaging, both indicators and sensors are employed to monitor the quality and safety of food products, yet they differ in their mechanism of action and outputs. Indicators are typically presented in the form of labels or tags and are designed to detect changes in environmental parameters such as pH, temperature, or gas composition. These changes are then transformed into visual responses, most commonly through color changes.^80^ Unlike sensors, indicators do not generate electronic signals, rather, they react directly with target analytes such as volatile amines, CO_2_ or other spoilage-related compounds.^78^ The response of an indicator can be either cumulative or threshold-based: cumulative if the indicator gradually changes over time as the spoilage condition progresses and threshold if becoming activated only when a critical limit is exceeded. For example, as microorganisms proliferate in food, they produce metabolic byproducts that can alter the pH of the environment. A pH-sensitive dye will change its color only when the acidity or alkalinity reaches a threshold value, thereby serving as a visual indicator of microbial spoilage. However, sensors are more advanced and active systems that are capable of detecting specific chemicals or biological analytes and converting them into measurable signals. Most sensors are made up of two basic components: a receptor and a transducer. The function of the receptor or sensing material is to interact with the target analyte and this interaction induces some physicochemical changes. The transducer then converts these changes into readable signals, typically an electrical, chemical, thermal, or optical signal.

### 3.1. Indicators

Indicators are devices incorporated within the food packaging that can provide information about the quality status of the food through some visual indications. Most indicators are based on colorimetric dyes, which produce different colors depending upon the presence or absence of target chemicals, the extent of reactions between two or more substances or the difference in the concentration of chemicals inside or outside the packaged food. Oxygen and carbon dioxide indicators, pH indicators, and time–temperature indicators are the most commonly used indicators in intelligent packaging.^81^

#### 3.1.1. Oxygen and carbon dioxide indicators

Even though food packaging is done under a controlled oxygen atmosphere such as modified atmospheric packaging (MAP),^82^ there are chances for oxygen leakage due to improper packaging or spoilage by trapped oxygen. Food spoilage by chemical oxidation is one of the major problems in the food industry. Some of the oxidative reactions responsible for food deterioration are the rancidity of unsaturated fats, pigment oxidation, and phenolic browning. The rancidity of unsaturated fats results in the production of toxic end products and off-flavors. Similarly, phenolic browning and pigment oxidation affect the color, taste, and, hence, the overall quality of the food.^83^ Moreover, chemical oxidation promotes the growth of aerobic bacteria and fungi. The use of oxygen indicators that can detect the presence of oxygen through color changes resulting from chemical or enzymatic reactions can be employed as an efficient tool for detecting the chemical oxidation of packed foods. Redox-based oxygen indicators function *via* oxidation-reduction reactions that cause visible color changes.^80^ These indicators typically use redox-sensitive dyes such as methylene blue or resazurin. In oxygen-rich environments, the dye is oxidized, resulting in a distinct color. For example, methylene blue appears blue and resazurin turns pink or blue depending on the degree of oxidation. In contrast, when oxygen is absent (anaerobic conditions), the dye remains in its reduced, colorless state. Owing to this clear and reversible color shift, redox indicators are widely used to monitor anaerobic conditions and detect potential spoilage in sealed food packaging.

**Fig. 6:**
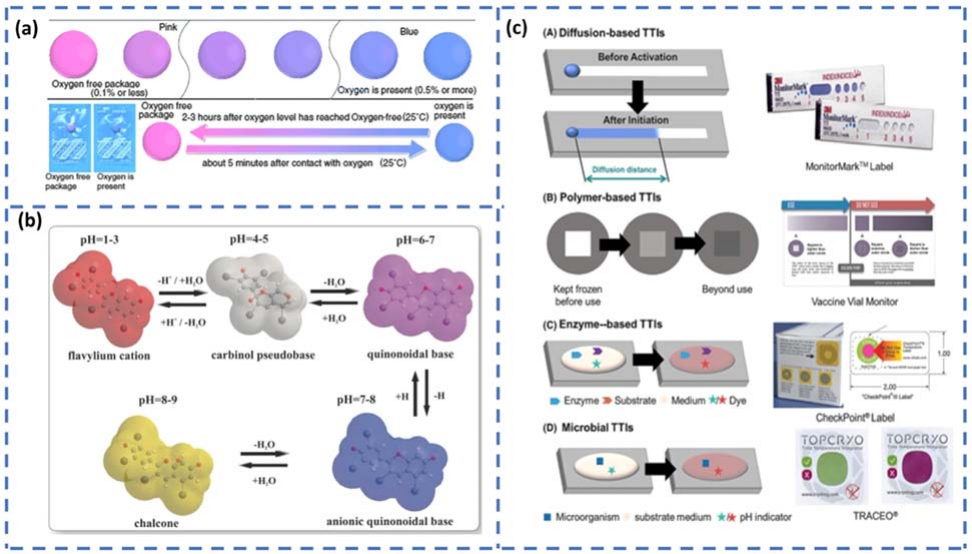
(a) Color pattern of the AGELESS EYE™ oxygen indicator [reproduced from ref. 85 with permission from Royal Society of Chemistry]. (b) Structure of anthocyanin at different pH values.^86^ (c) Classification of time–temperature indicators (TTIs) based on the working principle [reproduced with permission from ref. 87 Copyright 2023 John Wiley and Sons].

An example of a commercially available redox-based oxygen indicator is AGELESS EYE™. The color pattern of the indicator is given in Fig. 6a. Normally the color of the AGELESS EYE™ indicator will be pink. If the oxygen in the respective package gets depleted, the color of the indicator will change from pink to blue or purple. Once the oxygen in the package reduces, the indicator retains its original color. Won *et al.*^84^ developed a novel oxygen indicator based on natural components such as laccase, guaiacol and cysteine with in-pack activation. After physically separating these natural components into two compartments, the barrier separating them was breached. Upon rupture, the oxygen indicator was activated, undergoing color changes in response to the presence of oxygen. However, carbon dioxide (CO_2_) is generally a beneficial gas in food preservation, as it can inhibit microbial growth and extend the shelf life. In addition, CO_2_ is an inert flushing gas used during MAP for inhibiting microbial metabolism. Hence, any variation in CO_2_ concentration would result in food spoilage due to microbial/mold growth. Controlling CO_2_ levels is, therefore, crucial in maintaining the shelf life of food products. Thus, colorimetric oxygen and carbon dioxide indicators are key constituents in intelligent packaging, which is widely used to detect the amount of gas involved in food spoilage by means of a color change.

#### 3.1.2. pH indicator

Maintaining proper pH conditions is highly important for providing a good shelf life to the packed foods. However, the action of many microbial metabolites can affect the pH of the food environment. The change in pH is an indication of an alteration in product quality. Hence, the intelligent food packaging technique utilizes pH indicators or pH sensors to monitor the quality of packed foods. The pH indicators or pH sensors mainly consist of two parts, a solid matrix and a pH-responsive dye (natural or synthetic).^88,89^ The growth of microbes in food results in the release of a wide variety of metabolic byproducts such as H_2_S, NH_3_, CO_2_, organic acids, and other volatile substances. These released compounds will be adsorbed by the pH indicators attached to the package. The corresponding changes that occur in hydrogen or hydroxyl ion concentration cause structural alterations in the indicator.^90^ These changes cause a visible color change in the indicator and, hence, can be monitored easily. For example, anthocyanins are an interesting class of water-soluble flavonoid pigments found in fruits, vegetables, flowers, and leaves.^91^ Anthocyanins are responsible for the red-to-blue color of flowers and fruits. Besides, they have excellent antioxidant properties. The antioxidant activity and pH-responsive color changes of anthocyanins are mainly dependent on their molecular structure having an ionic nature. Anthocyanins have a red color at acidic pH and they shift to blue in the basic environment.^92^ These color effects arise from the variations in ionic states and electronic configurations within the molecule. In a strongly acidic environment, anthocyanins predominantly exist as red flavylium cations. As the pH increases, these cations undergo structural degradation and rapid hydration at the C-2 position, forming a colorless carbinol pseudobase, leading to a fading of the red color. Under neutral to alkaline conditions, anthocyanins primarily convert into purple and blue quinoidal base structures, which eventually transform into pale-yellow chalcone forms^86^ (Fig. 6b). Generally, fresh meat and fish products have a pH in the range of 5.5 to 6.2.^93^ Ammonia, amines and other basic volatile compounds released during amino acid breakdown shift the pH to higher values. Similarly, organic acids generated during glucose fermentation will also increase the pH value. Hence, if pH indicators are used, consumers can easily identify the changes in food quality through the color change of the indicator.

#### 3.1.3. Time–temperature indicators (TTI)

Time–temperature indicators are used for monitoring the temperature history of perishable food products, thereby providing information regarding their quality and shelf life throughout their storage, distribution, and consumption.^94^ Consumers are made aware of the current food quality status *via* an irreversible color change that occurs in TTI according to the temperature fluctuations of the packaged food. Based on the capabilities, TTIs are classified into three categories.^95^ This includes critical temperature indicators, partial history indicators, and full history indicators. The critical temperature indicators are those that indicate whether a product is exposed to above or below the permitted temperature. Partial history indicators indicate that, whether the food is exposed to a temperature enough to cause a chemical change affecting food quality. However, full history indicators record the complete temperature history of food throughout its supply chain. Based on the working principle, TTIs are classified into four major categories^87^ (Fig. 6c). This includes (a) diffusion-based, (b) polymer-based, (c) enzyme-based, and (d) microbial-based TTIs. Diffusion-based TTIs operate through temperature-dependent diffusion or phase transitions of colored ester dyes such as butyl stearate or dimethyl phthalate. These dyes start diffusion through a channel (often a capillary fiber) once the temperature exceeds their melting point. The diffusion rate increases with temperature, and the resulting diffusion distance indicates the combined effect of time and temperature. Typically, these TTIs are activated by pressing to initiate dye contact and start the response. Polymer-based temperature indicators are chemical markers that exhibit color changes by solid-state polymerization, generally employing diacetylene derivatives. Upon exposure to heat or radiation, these compounds undergo 1,4-addition, resulting in the formation of polydiacetylenes (PDA), which induces an observable color shift related to alterations in the conjugated backbone. Elevated temperatures accelerate the process, resulting in more rapid color transitions, thus enhancing their efficacy for monitoring temperature exposure over time. Enzyme-based TTIs rely on temperature-sensitive reactions between specific enzymes and substrates. These reactions cause pH changes that alter the color of a dye in the medium, indicating food quality and safety. The wide range of enzyme-substrate pairs allows these TTIs to be tailored for monitoring various types of foods. For example, the enzyme lipase breaks down a lipid substrate into free fatty acids, leading to a pH decrease that is detected by an appropriate pH indicator dye. Microbial TTIs imitate real spoilage by employing microbial growth and metabolism to trigger color changes over time and temperature. As microorganisms metabolize, they modify the pH of the medium, resulting in a visible and irreversible color change in a pH indicator. These TTIs generally comprise a microorganism, a nutrient medium, and a pH-sensitive dye, enabling them to accurately represent product quality variations associated with time–temperature exposure. Some recently developed indicators for intelligent food packaging are summarized in Table 3.

**Table 3:** Some recently developed colorimetric pH indicator films

Indicator	Analyte	Food	Polymer matrix	References
Lysine/ε-polylysine/anthocyanin	CO_2_	Poultry meat	PET	96
Anthocyanin/limonene	*Bacillus subtilis*, *Aspergillus niger*, and *Staphylococcus aureus*	Pasteurized milk	Starch/PVA	97
Anthocyanin–ovalbumin-CMC nanocomplex	CO_2_	Mushroom	Corn starch/PVA	98
Curcumin/anthocyanin	Volatile amines	Fish	Starch/PVA	99
Red radish anthocyanin	Volatile amines	Fish	Gelatin/gellan gum	100
	Organic acids	Milk		
Anthocyanin/propolis extract	*Escherichia coli* and methicillin-resistant *Staphylococcus aureus*	Pasteurized milk	PVA/starch	101
Black rice bran anthocyanin/oregano essential oil	Ammonia	Pork	Chitosan	102
Alizarin/grapefruit seed extract	Volatile gas		CMC/agar	103
Anthocyanin	Ammonia	Pork	Starch/carbon dots	104
	Microbes			

### 3.2. Sensors

Sensors are devices that detect, locate, or quantify a problem and then send signals to measure its physical or chemical characteristics. Sensors can provide continuous output signals in response to changes in the surrounding environment. Most sensors are made up of two basic components: receptor and transducer. The function of the receptor or sensing material is to interact with the target analyte and to convert the resulting physical or chemical information into the form of energy. This energy will then be converted by the transducer into a readable signal, typically an electrical, chemical, thermal, or optical signal.^105^ Depending upon the response stimuli, the sensors are of two different types: gas sensors and bio-sensors.

#### 3.2.1. Gas sensors

Gas sensors, as the name suggests, are employed for the detection of gaseous analytes present inside the package. Commonly available gas sensors include oxygen (O_2_) sensors, carbon dioxide (CO_2_) sensors, humidity/water vapor sensors, basic nitrogen compound sensors, and ethanol (alcohol-based) sensors. Other than these, piezoelectric crystal sensors, metal oxide semiconductor-based sensors, and organic conducting polymer sensors are also known.

**Fig. 7:**
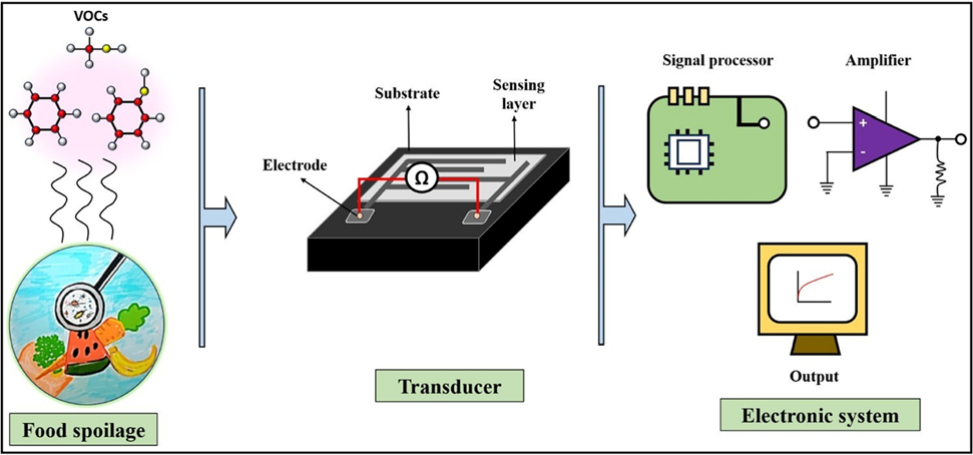
Schematic depicting the gas sensing process in food quality monitoring.

The microbial decomposition of nutrients in food products can lead to the emission of various volatile organic compounds (VOCs) including amines (such as ammonia, dimethylamine, and trimethylamine), hydrogen sulfide (H_2_S), carbon dioxide (CO_2_), oxygen (O_2_), ethylene (C_2_H_4_), and aldehydes and ketones. These compounds serve as indicators of spoilage and alter the food quality. The gas sensors can detect and quantify the concentrations of these VOCs, thus enabling the real-time monitoring of food freshness.^106^ Thus, incorporating gas sensors to food packaging can provide consumers and suppliers with immediate information regarding the condition of the food, thereby enhancing food safety and reducing waste. Fig. 7 illustrates the gas sensing mechanism in food quality monitoring.

As already mentioned in Section 3.1.1., CO_2_ is generally accepted as an active packaging gas that can minimize the oxygen content inside the food package to inhibit the growth of aerobic microbes, thereby extending the shelf life of food products. Therefore, food deterioration can be directly determined from the CO_2_ concentration. For example, conventional CO_2_ sensors can be broadly classified into two types, based on the type of transducer: optical and electrochemical CO_2_ sensors. Optical sensors can be further classified into colorimetric CO_2_ sensors and fluorescent CO_2_ sensors. Both the fluorescent and colorimetric CO_2_ sensors detect carbon dioxide *via* chemical interactions between carbon dioxide and the sensing materials, but they differ significantly in their detection mechanisms and practical applications. Fluorescent CO_2_ sensors utilize fluorescent dyes or probes that respond to CO_2_ by exhibiting changes in their fluorescence properties such as intensity, wavelength, or lifetime.^107^ Typically, CO_2_ reacts with water in the sensor matrix to form carbonic acid (H_2_CO_3_), which alters the pH and consequently affects the fluorescence of pH-sensitive dyes. In some systems, CO_2_ directly interacts with amine groups on the fluorescent molecules, forming carbamate species that cause fluorescence quenching or spectral shifts. These changes are detected by exciting the sensor with UV or visible light and measuring the emitted fluorescence, enabling highly sensitive and quantitative detection of CO_2_ at low concentrations.^108^ However, colorimetric CO_2_ sensors operate through a visible color change caused by similar chemical interactions, where CO_2_ dissolves in water to produce carbonic acid, lowering the pH and triggering a color transition in pH-sensitive dyes such as bromothymol blue or phenol red.^109^ Unlike fluorescent sensors, colorimetric sensors provide a simple, low-cost, and power-free way to monitor CO_2_ levels that can often be interpreted by the naked eye, but with lower sensitivity and less precise quantification. Fluorescent sensors require instrumentation for excitation and emission detection but offer faster response times, higher sensitivity, and better suitability for real-time and quantitative applications.

Bigiani *et al.*^110^ have developed a MnO_2_-based ethylene gas sensor for monitoring fruit ripening. The fabrication was carried out by a two-step plasma-assisted process, where the first step is the chemical vapor deposition of MnO_2_ on the polycrystalline Al_2_O_3_ substrate followed by functionalization with Ag and Au nanoparticles by a sputtering process. This composite has exhibited high sensitivity towards ethylene gas even at a very low ppm range.

#### 3.2.2. Biosensors

**Fig. 8:**
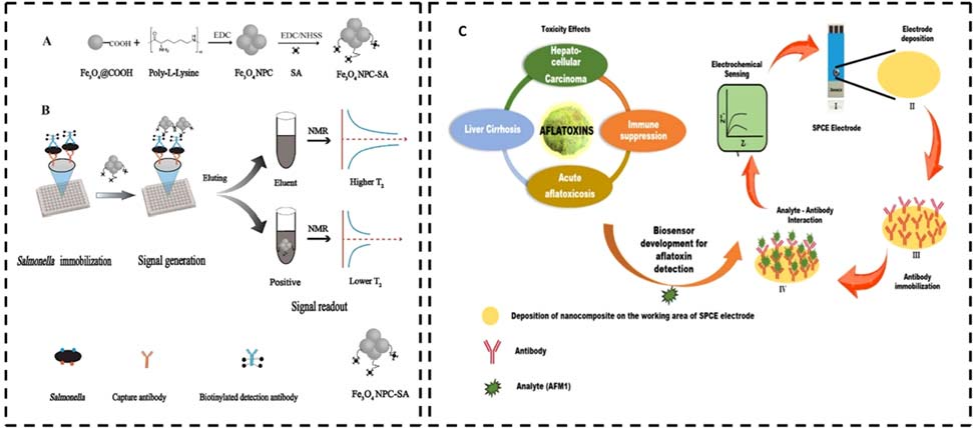
(A) Preparation of Fe_3_O_4_ NPC and Fe_3_O_4_ NPC-SA. (B) Outline of the NMR biosensor for *Salmonella* detection in milk [reprinted with permission from ref. 112 Copyright Elsevier 2019]. (C) Electrochemical sensor based on the MoS_2_/UiO-66-NH_2_ composite for AFM1 detection [Copyright © 2022, American Chemical Society].

Biosensors can be used to analyse and detect natural toxins, antinutrients, and food spoilage pathogens present in food packages through enzymatic and immunogenic reactions. Compared to gas sensors, here the receptors are made up of biological materials such as enzymes, antigens, hormones, or nucleic acids. The transducer then converts this biological recognition into measurable signals. Depending on the measuring parameter, the transducer can be of electrochemical (amperometry, voltammetry, and potentiometry), optical, acoustic, or thermal nature.^111^ Biosensors have diverse applications in ensuring food safety and security, including the detection of foodborne pathogens, natural toxins, veterinary drug residues, pesticide traces, and various chemical contaminants such as food allergens and heavy metals. Foodborne diseases are primarily caused by a wide range of pathogenic organisms such as *Salmonella* spp., *Campylobacter* spp., and enterohaemorrhagic *Escherichia coli*. Salmonella infection or salmonellosis is associated with the consumption of infected eggs, poultry, and other animal-derived items. Campylobacter, a prominent source of bacterial foodborne illness usually comes with raw milk, undercooked or raw chicken, and contaminated drinking water. A novel nuclear magnetic resonance (NMR) biosensor was developed for the sensitive detection of *Salmonella* in milk, utilizing Fe_3_O_4_ magnetic nanoparticles (NPs) as probes^112^ (Fig. 8A and B). To enhance the sensitivity, signal amplification strategies such as the streptavidin-biotin system and the formation of magnetic nanoparticle clusters (NPCs) were employed. These NPCs, formed by conjugating individual Fe_3_O_4_ NPs with poly-l-lysine (PLL), exhibited a collective effect that significantly reduced the transverse relaxation time (*T*_2_), thereby improving the detection efficiency. The biosensor demonstrated a limit of detection (LOD) of 10^5^ CFU mL^−1^ in milk samples. Similarly, aflatoxins are highly carcinogenic hepatotoxins produced by fungi such as *Fusarium*, *Aspergillus*, and *Penicillium* with serious health risks. Aflatoxin M1 (AFM1), primarily secreted in the milk of mammals consuming AFB1-contaminated feed, poses serious health risks such as liver damage, weakened immunity, and higher infections. A novel and sensitive electrochemical sensor for detecting aflatoxin M1 (AFM1) was developed by modifying screen-printed carbon electrodes with a nanocomposite of MoS_2_ quantum dots and UiO-66-NH_2_, a zirconium-based metal–organic framework.^113^ Monoclonal antibodies specific to AFM1 were immobilized on the modified electrodes (Fig. 8C). Using electrochemical impedance spectroscopy, the sensor achieved a detection range of 0.2–10 ng mL^−1^ with a low detection limit of 0.06 ng mL^−1^Table 4 lists some of the commercially available biosensors.

**Table 4:** List of commercially available biosensors^114^

Target analyte	Company	Country
Ethanol, methanol, glucose, lactate, glycerol	Analox Instruments	UK and USA
*E. coli* in lettuce	Massachusetts Institute of Technology	USA
Proteins, toxins, viruses, bacteria, spores and fungi	Research International	USA
Atrazine	Universitat Autonoma de Barcelona in collaboration with CSIC	USA
Allergens, vitamins, microorganisms	Biotech-lgG	Sweden
Amines	Oriental electric	China

One of the drawbacks associated with these sensors is their selectivity. Real food systems are highly complex, and hence, potential interference from various components in the food matrix such as other volatile organic compounds (VOCs), moisture, and fat can affect the accuracy and reliability of indicator responses. For example, if a sensor is particularly designed to detect a specific spoilage analyte like hydrogen sulfide or ammonia, they may exhibit cross-reactivity with other volatiles naturally present in the food matrix. This can lead to a false-negative response. Quartz crystal microbalance (QCM) biosensors are effective for the detection of pathogens and other quality indicators. However, their sensitivity can be influenced by food components such as fats and proteins. These substances may compete with the target analyte binding on the sensor surface, thus minimizing the accuracy.^115^

### 3.3. Data carriers

Data carriers are automatic recognition devices incorporated within the food packages that store and transmit information in the food supply chain.^116^ Data carriers are not intended to provide any information regarding the food quality status; instead, they are utilized for traceability, automatization, theft prevention, or counterfeit protection. They are mostly placed onto tertiary packaging systems such as large cardboard boxes, pallets, and multi-box containers. Barcode labels and radio frequency identification devices (RFID) are the most common data carriers used in the food packaging industry.^117^

#### 3.3.1. Bar codes

Bar codes are the most popular and least expensive form of data carriers. They store data in the form of spaces and parallel lines that can be scanned or read by optical scanners and smartphones. Barcodes on food packaging are an excellent way to track, monitor, and also to guarantee that every single item which enters the supply chain is of the highest possible quality. There are two main types of barcodes: one-dimensional (1D) and two-dimensional (2D) barcodes. The 1D barcode includes a pattern of bars and spaces to represent 12 digits of data, providing information regarding the manufacturer identification number or item number,^118^ whereas the 2D barcode offers more storage capacity than 1D and it allows the transfer of data up to 1.1 kilobytes. ‘Quick response code’ (QR code) and ‘data matrix’ are the two main standardized 2D barcodes.^119^ It enables the encoding of additional information that is not possible with linear barcodes such as nutritional information, cooking recipes, and even access to the manufacturer's website.

#### 3.3.2. Radio frequency identification (RFID) tag

RFID tag is an advanced form of data carrier for automatic product identification and traceability. It can store data up to 1 MB. Inside a RFID tag, a microchip is connected to a tiny antenna that has been printed or etched onto a paper material or on polymer films such as polyethylene terephthalate (PET). These RFID tags can then be attached to the products.^120^ In conventional RFID systems, radio frequency waves are emitted from the reader and the emitted waves are captured by the RFID tag. The captured waves are then transferred to a computer for further analyses. Many studies were conducted on RFID tags for temperature, humidity, gas, pH, and traceability sensor applications in connection with food packaging.^121–123^

## 4. Electrospun membranes for smart food packaging applications

Electrospinning is a simple and highly versatile technique used for developing smooth and thin fibrous membranes with high surface area and porosity. Due to its unique surface characteristics, electrospun membranes are widely used in membrane-based filtration, sensing, water treatment, energy storage, and biomedical applications.^124,125^ In the food industry, electrospun membranes are extensively used as novel platforms for smart food packaging. Since these membranes possess tunable surface characteristics, they can be easily functionalized by incorporating active components. These encapsulated active components can ensure sustained and controlled release from the membranes due to the inherent porosity and fibrous nature associated with the electrospun membranes.

Electrospun membrane porosity plays a crucial role in controlling the respiration rates and moisture levels in perishable food packaging, especially in the case of fresh fruits and vegetables (FFV).^126^ As mentioned in Section 2.2, FFV's are easily susceptible to postharvest loses mainly due to transpiration and condensation. The interconnected porous structure of electrospun membranes allows for selective gas permeability, which can be tailored to match the respiration rates of specific fruits and vegetables. By optimizing the pore size and distribution, these membranes can regulate gas exchange to effectively slow down the ripening process and extend the shelf life. Additionally, the high surface area-to-volume ratio facilitates interfacial interactions with gases, providing selective barrier properties against water vapor, oxygen, and other environmental gases by creating a long and tortuous diffusion path, which enhances gas and moisture resistance compared to conventional packaging materials. This selective permeability not only prevents excessive moisture accumulation that could foster microbial growth, but also minimizes excessive moisture loss that can cause food dehydration. Thus, tuning the membrane porosity *via* customized packaging solutions can be achieved that meets specific requirements of perishable foods.

**Fig. 9:**
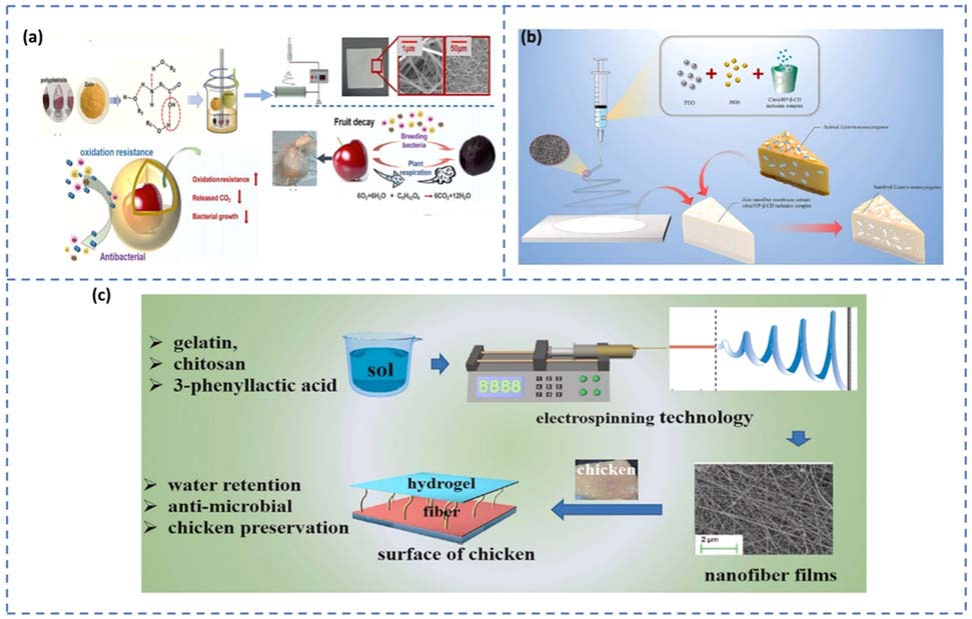
(a) Schematic showing the fabrication and application of electrospun zein/gelatin films with polyphenols for cherry preservation [reprinted with permission from ref. 127 Copyright Elsevier 2023]. (b) Zein nanofiber membrane encapsulated with an HP-β-CD inclusion complex for cheese preservation [reprinted with permission from ref. 128 Copyright Elsevier 2022]. (c) Antibacterial gelatin/chitosan/3-phenyllactic acid for extending the shelf life of chilled chicken meat [reprinted with permission from ref. 129 Copyright Elsevier 2022].

Recently, electrospun-blended nanofibers composed of zein and gelatin, enriched with natural polyphenols, were used as active packaging materials for extending the shelf life of fresh cherries (Fig. 9a).^127^ These biopolymer-based nanofiber mats demonstrated high hydrophobicity, which effectively minimized moisture loss from the fruit during storage. This moisture retention was evidenced by reduced firmness loss, lower respiratory activity, and decreased ethylene production in the cherries when stored at 20 °C for up to 12 days. In dairy product packaging, the high porosity enables the incorporation of antimicrobial agents and antioxidants that help extend the shelf life. Zein-based nanofibers embedded with an inclusion complex of citral and hydroxypropyl-β-cyclodextrin (HP-β-CD) demonstrated favourable characteristics for active food packaging, especially in cheese preservation (Fig. 9b).^128^ The addition of zein improved the tensile strength, thermal stability, and hydrophobicity of the nanofibers, making them suitable for moisture-sensitive applications. Moreover, the porous structure of the nanofiber matrix enabled a prolonged release of citral, a bioactive agent known for its antibacterial properties. This controlled release facilitated the preservation of cheese by preventing the growth of *Listeria monocytogenes* and prolonging its shelf life under the storage conditions of 4 °C and 12 °C. An effective antibacterial hydrogel was developed using electrospun nanofibers composed of gelatin, chitosan, and 3-phenyllactic acid, for extending the shelf life of chilled chicken meat (Fig. 9c).^129^ The distinctive structural characteristics of electrospun nanofibers, including their uniform and dense network, high surface area, effective entrapment of pathogens, and controlled release of active compounds render them highly suitable for food preservation applications. Upon absorbing moisture, the nanofibers rapidly transformed into a hydrogel, which demonstrated significant antimicrobial activity against foodborne pathogens and successfully prolonged the shelf life of chilled chicken by up to four days.

In order to demonstrate the superiority of electrospun nanofiber membranes over solventcast films, Turanli *et al.*^130^ conducted a comparative study. In this study, polyvinyl alcohol (PVA/chitosan (CS) blend materials were fabricated *via* electrospinning and solvent casting. This comparative analysis demonstrated enhanced performance of electrospun fibers in terms of thermal stability, antioxidant activity, and adsorption capacity, attributed to their higher surface area, porosity, and fiber alignment. Despite their improved thermal stability, electrospun samples showed lower glass transition (*T*_g_) and melting temperatures (*T*_m_), probably due to enhanced chain mobility from their porous architecture. A comparative summary of the obtained values is presented in Table 5.

**Table 5:** Comparative analysis of PVA/CS blend films fabricated *via* electrospinning and solvent casting^a^

Sample	Water contact angle (°)	*T*_g_ (°C)	*T*_m_ (°C)	Radical scavenging activity (%)	
				DPPH	ABTS
CF-PVA-CS	43 ± 1	80	225	28.8	22.8
NF-PVA-CS	40 ± 1	74	223	38.9	34.6

a CF = solvent cast, NF = nanofiber.

**Fig. 10:**
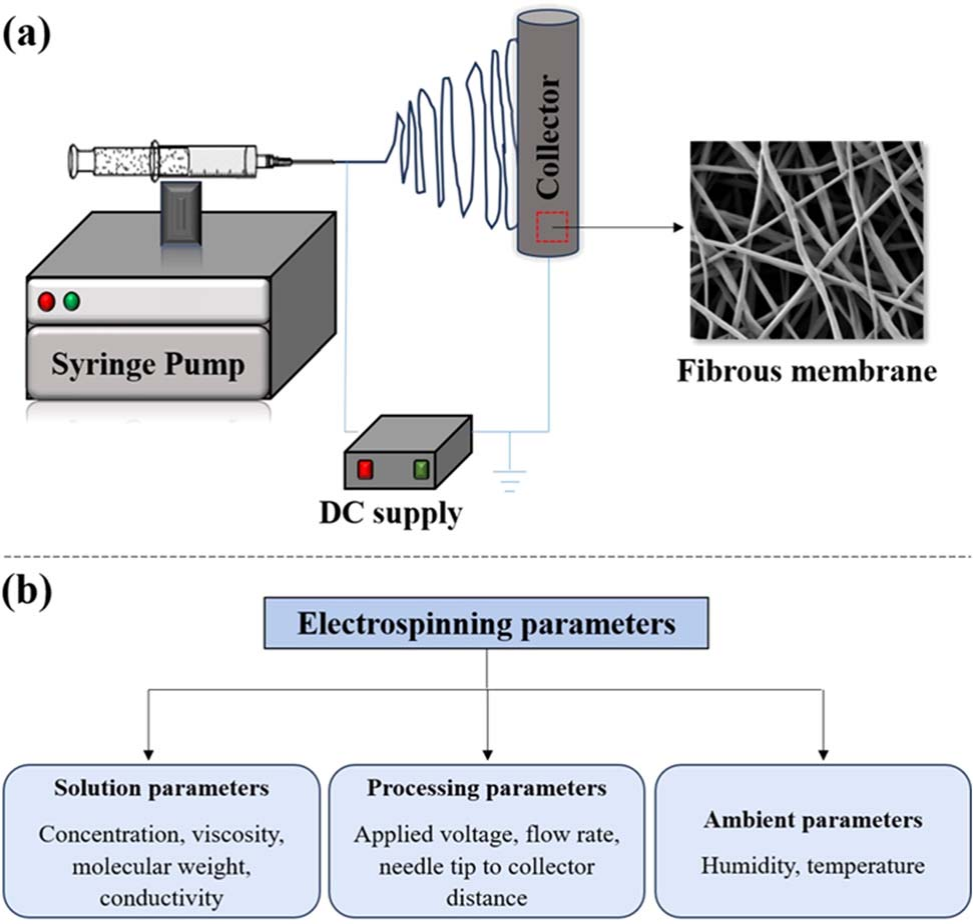
(a) Schematic of a basic electrospinning setup with syringe pump, high voltage supply and collector systems. (b) Outline of various electrospinning parameters.

In electrospinning, the production of continuous nanofibers from the polymer solution or melt is done with the application of high voltage.^131^ This is achieved with the help of a high-voltage supply system, a syringe pump system, and a grounded collector. The schematic of a basic electrospinning unit is shown in Fig. 10a. The first step of the process involves polymer solution preparation. The prepared solution can be then taken in a suitable syringe system equipped with a metallic needle. The next step involves proper fixing of the solution-filled syringe in the syringe pump setup. Once all these initial processes are done, a high voltage is applied between the tip of the metallic spinneret and the collector. The application of electric field results in the charging of the polymer solution. As the process progresses, there occurs charge repulsion, and hence, the solution gets extruded through the metallic spinneret as a liquid drop. Once the electric field intensity is sufficient enough to overcome the surface tension, the liquid droplet deforms into a conical shape known as the *“Taylor cone”.* When the electric field is increased further, the electrostatic repulsive force overcomes the surface tension, and the liquid jet is ejected out. The ejected liquid jet is then directed towards the metallic collector. During this time, the liquid jet initially extends in a straight line. However, due to bending instabilities and lengthening, the straight jet undergoes vigorous whipping motion. This whipping motion allows the polymer chains within the solution to stretch and slide past each other, resulting in the formation of fine fibers with a diameter in the nanoscale range. Finally, the excess solvent in the formed fibers get evaporated, and solid fibers are deposited onto the collector surface.^132^

### 4.1. Electrospray-electrospinning

**Fig. 11:**
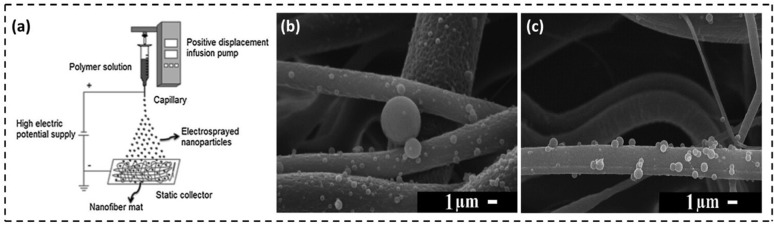
(a) Schematic showing electrospraying of nanoparticles over static collectors containing nanofibers. (b) and (c) SEM images of PCL and PLLA nanofibers coated with PC-PVA nanoparticles [reprinted with permission from ref. 135 Copyright Elsevier 2019].

Electrospray-electrospinning is an emerging technology that combines two complementary techniques, namely electrospraying and electrospinning, to fabricate advanced functional materials. Electrospraying is a variation of the electrospinning technique that creates micro/nanoparticles through the hydrodynamic atomization of a polymer solution when exposed to a strong electric field.^133,134^ This method has received significant attention in food packaging applications for the development of films or bioactive coatings that impart distinct properties to the final product. Although bioactive compounds offer significant benefits in food packaging, they often lose their functionality when exposed to organic solvents in polymer solutions for extended periods. Electrospraying provides an effective strategy to overcome this limitation by enclosing sensitive compounds within polymeric nanoparticles. This protective coating preserves their biological activity and shields them from environmental stresses such as fluctuations in pH, temperature, humidity, and light. Schmatz *et al.*^135^ developed an innovative nanocomposite material consisting of polycaprolactone (PCL) or poly-l-lactic acid (PLLA) nanofibers coated with phycocyanin-incorporated polyvinyl alcohol (PVA) (PC-PVA) nanoparticles, using electrospinning and electrospraying techniques. Fig. 11a demonstrates the electrospray–electrospinning process along with the SEM images of PCL nanofibers (Fig. 11b) and PLLA nanofibers (Fig. 11c) coated with PC-PVA nanoparticles, respectively. The resulting nanomaterial exhibited favorable mechanical strength (31 ± 7 to 228 ± 26 kPa) and thermal stability (285–366 °C), effectively protecting phycocyanin from mechanical stress and thermal degradation. The direct deposition of nanoparticles onto the nanofiber surface mitigates challenges related to nanoparticle recovery from the collector. A significant advantage of this method is the immobilization of phycocyanin within the interstitial spaces of the polymeric matrix, facilitating a more controlled and sustained release of the bioactive compound upon contact with the target food. This prolonged release enhances antioxidant activity and aids in preserving the sensory qualities of the packaged food.

### 4.2. Parameters affecting fiber characteristics

Adjusting electrospinning parameters is essential for customizing nanofiber characteristics to satisfy the specific requirements of food packaging applications, especially in the context of smart and active packaging systems. Adjusting parameters including polymer concentration, applied voltage, solution flow rate, tip-to-collector distance, and solvent composition allow for the precise control over fiber morphology, which influences the diameter, porosity, surface roughness, and alignment. The structural features directly impact functional performance. Smaller fiber diameters and increased porosity contribute to an enhanced surface area, facilitating improved interaction with embedded sensors or bioactive compounds, which is crucial for responsive packaging. Optimizing surface roughness enhances the adhesion and visibility of colorimetric indicators. Additionally, modulating hydrophilicity influences moisture sensitivity and barrier properties. Strategic tuning of electrospinning parameters ensures uniform and stable fiber formation, enabling the design of packaging membranes with tailored functionalities for real-time monitoring, extended shelf life, and improved food safety.

There are three main parameters that influence the overall fiber characteristics including fiber diameter, fiber morphology, and fiber distribution.^136^ These include the processing, solution, and environmental parameters (Fig. 10b).

#### 4.2.1. Processing parameters

An important processing parameter is applied voltage. Generally, current from the applied voltage is responsible for the transformation of spherical droplets into *“Taylor cone”*, and finally, the formation of ultrathin nanofibers. Therefore, applied voltage plays a vital role in fiber morphology.^137^ Nanofiber formation occurs only when the applied voltage exceeds a certain critical value depending on the polymeric solution. As the applied voltage is increased, nanofibers with small diameters are formed due to the stretching of the polymer solution, which can be attributed to the increased charge repulsion within the polymer jet. However, there are different arguments regarding the effect of applied voltage on the electrospinning process. Some studies suggest that higher voltages lead to increased ejection of the polymer solution, resulting in fibers with larger diameters. Conversely, other reports indicate that elevated voltages may produce non-uniform fibers with bead formation.

The next process parameter is flow rate. Flow rate is defined as the amount of polymer solution flowing out of the needle tip to the *Taylor cone* per unit time. The flow rate can influence the droplet size, stability of the *Taylor cone*, trajectory of the jet, fiber diameter, and fiber distribution.^138^ Every polymer solution possesses a critical flow rate at which uniform beadless nanofibers are generated. At lower flow rates, the solvent will get enough time for evaporation, generating fibers of smaller diameter. However, increasing the flow rate above a critical value will result in beaded and thicker fibers with larger diameters. This is because of the insufficient drying time for the evaporation of the solvent and the low stretching force of the solution during the time of flight between the needle and the collector. A study conducted by Zargham *et al.*^139^ on electrospun nylon 6 nanofibers showed that the *Taylor cone* becomes more stable at a low flow rate of 0.5 mL h^−1^, and at this low flow rate, fibers with the narrowest fiber distribution were obtained. However, at higher flow rates, some defects such as fiber splitting, branching, and formation of web-like structures were observed due to insufficient solvent evaporation.

The distance between the needle tip and the collector is also considered as an important processing parameter. If the distance is too short, the fibers will not get enough time for solvent evaporation and proper stretching. This results in the formation of beaded fibers with higher diameters. Similarly, if the distance is too long, very thin fibers with small diameters are observed. Therefore, an optimum distance must be maintained for getting fibers with appropriate fiber characteristics. Recently, Mariola *et al.*^140^ have studied the influence of processing parameters such as distance, voltage, and flow rate on the morphology and diameter of electrospun polyvinyl alcohol (PVA) fibers. The fiber diameter was analyzed by varying the tip-to-collector distance: 10 cm; 836 ± 301 nm, 15 cm; 814 ± 309 nm, 20 cm; 727 ± 218 nm. A minimum fiber diameter was observed at a distance of 20 cm, which can be explained by two effects that occurred simultaneously causing opposing tendencies. That is, if the tip-to-collector distance is increased, the intensity of the electric field will decrease, forming a less stretched polymer solution with thicker fibers. At the same time, long distances will provide enough time for solvent evaporation forming thinner fibers.

#### 4.2.2. Solution parameters

The solution concentration is an important parameter in the electrospinning process. There will be a critical solution concentration for each polymer at which uniform beadless electrospun fibers can be produced. Any concentration change from this critical value will result in fibers with a defective morphology.^141^ At low solution concentrations, the applied electric field and surface tension causes the highly entangled polymeric chains to break into fragments, thus instead of electrospinning, electrospraying will occur.^142^ However, an increase in polymer concentration will hinder the flow of solution through the needle tip, which ultimately yields defective or beaded nanofibers. Tao *et al.*^143^ reported that the structure of electrospun PVA fibers (*M*_w_ = 8000 g mol^−1^) changed from bead (16%) to beaded fibers (18%), then to smooth fibers (22%), and finally, flat ribbons (27%), as the concentration is increased at a constant molecular weight.

The molecular weight of the polymer is another important factor, as it influences other factors such as viscosity, surface tension, conductivity, and dielectric strength of the polymer solution.^144^ Generally, in electrospinning, high molecular weight polymers generate smooth fibers with uniform morphology than low-molecular-weight polymers.

Solution viscosity is another important factor in the electrospinning process.^145^ At very low viscosity, continuous and smooth fibers cannot be produced and at very high viscosity, the polymer jet cannot be easily discharged from the needle tip. The polymer concentration and molecular weight are closely related to the respective solution viscosity. Hence, by adjusting these two parameters, a solution with proper viscosity that can produce optimum fiber morphology can be made. Nezarati *et al.*^146^ investigated the effect of solution viscosity on the fiber morphology by mixing polycarbonate urethane (PCU) in *N*,*N*-dimethylacetamide (DMAc) at different concentrations. Generally, as the solution concentration increases, the solution viscosity also increases. It was observed that beaded fibers were formed at a low viscosity (7.2 ± 1.7 Pa s), uniform fibers at an intermediate viscosity (10.1 ± 0.5 Pa s), and fibers with a large diameter at a higher viscosity (22.5 ± 1.4 Pa s).

Another important solution parameter influencing the fiber morphology is the surface tension. For the initiation of the electrospinning process, the charged solution needs to overcome surface tension. It has been reported that a lower surface tension can produce beadless fibers with smaller diameters, whereas a higher surface tension leads to instabilities in the polymer jet and thus produces fibers with non-uniform morphology. Surface tension is strongly dependent on the nature of the solvent used for making the polymer solution. Yang *et al.*^147^ have investigated the effect of solvents with different surface tensions (DMF, ethanol, and DCM) on the morphology of polyvinyl pyrrolidone (PVP) electrospun fibers. A single solvent system with 4 wt% PVP concentration in ethanol displayed a smooth fibrous texture, in contrast to those produced using DMF and DCM, which showed a beaded morphology. Within mixed-solvent configurations, the combination of ethanol and DMF at a 50/50 mass ratio provided fine PVP fibers with a very small diameter of 20 nm. Therefore, solvent selection is an important criterion in the electrospinning process.^148^

The electrospinning process is mainly dependent on the Coulomb forces between charges on the polymer jet surface and electrostatic force due to the external electric field.^149^ The repulsion between these surface charges is responsible for the stretching and formation of nanofibers. Surface charge interactions can thus be studied by altering the solution's conductivity. The solution conductivity not only affects the formation of the *Taylor cone*, but also influences the fiber diameter. A significant drop in nanofiber diameter has been observed with the increase in electrical conductivity due to the accumulation of more charges on the jet.^150^ Conductivity can be altered by adding salts, ions or conducting polymers to the electrospinning solution.^151^ Angammana *et al.*^149^ conducted a detailed study on the polyethylene oxide (PEO)/water system to investigate the effect of solution conductivity on the entire electrospinning process and fiber morphology by the addition of a NaCl salt. The group initially observed an increase in average jet current with the increase in conductivity and then it gradually declined. However, the average fiber diameter decreased with an increase in conductivity. They also found out that polymer solutions with low conductivity cannot undergo an electrospinning process, as there is no charge on the liquid surface to form the *Taylor cone.*

#### 4.2.3. Environmental parameters

Temperature can influence both the viscosity and the solvent evaporation rate of polymer solutions in an opposing way. That is, the evaporation rate of the solvent decreases exponentially with the decrease in temperature. However, at higher temperatures, the solution viscosity decreases due to the increased freedom of movement of polymer chains.^152,153^ Reduction in both the solvent evaporation rate and the solution viscosity results in thinner fibers. A study was conducted by Mahdi *et al.*^154^ on the influence of temperature on the morphology and diameter of polyacrylonitrile (PAN) nanofibers. In this study, when the temperature was varied from 20 °C to 30 °C, uniform bead-free fibers were obtained at higher temperatures. Moreover, the average diameter of PAN electrospun nanofibers decreased from 167 nm to 118 nm within this temperature range.

Similarly, variations in humidity can also influence the fiber morphology in terms of pore size, its number, and fiber diameter. Ramakrishnan *et al.*^155^ studied the effect of humidity on electrospun polycaprolactone (PCL) nanofibers for drug delivery applications. When the humidity factor was increased from 40% to 60% RH, an increase in fiber diameter from 100 nm to 145 nm was observed. The fibers produced were also more uniform at 60% relative humidity. It was also reported that, at high humidity, small circular pores can be formed at the fiber surface.^156^ If the humidity is too low, the solvent evaporation will be faster causing the solution near the needle tip to be clogged, hence preventing the proper electrospinning process.

### 4.3. Biopolymers suitable for electrospinning and their applications in smart food packaging

**Fig. 12:**
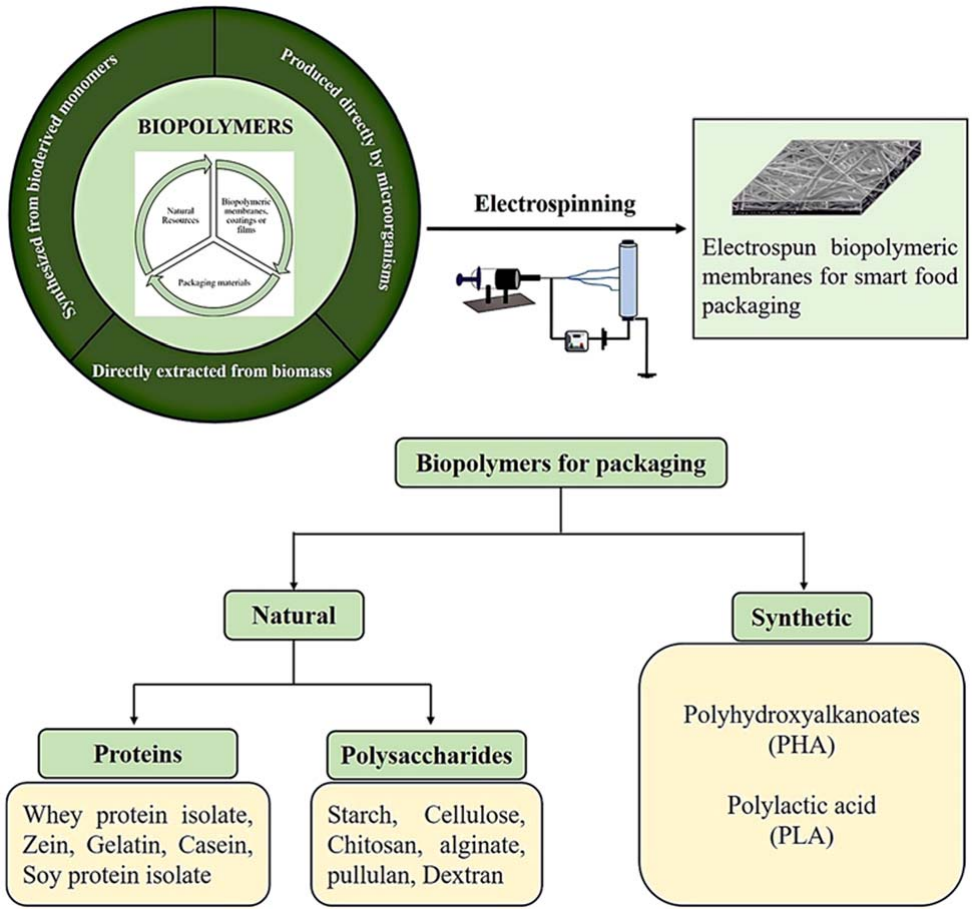
Schematic outline of biopolymers and their types used for packaging applications.

The smart food packaging materials can offer many additional benefits over the traditional packaging systems. The highly porous structure and large surface area-to-volume ratio of electrospun fibers make them an ideal platform for fabricating multifunctional nanostructured films for food packaging applications. In the food industry, electrospun nanofibers can be used in many ways such as reinforcing fillers and structural components in food matrixes, and for the encapsulation of bioactive substances such as antimicrobials, antioxidants, enzymes and vitamins.^157^ These nanofibers can protect the food from oxidation, moisture, and light. Additionally, it facilitates the release of bioactive compounds and improves the viability of probiotics. By combining proteins and polysaccharides, these fibers provide an environmentally friendly, biodegradable, and cost-efficient packaging solution using materials such as starch, zein, gelatin, PHB, PHBV, and PLA.^158^ For food-related applications, the material used has to be safe and biocompatible. In this regard, natural polymers are safer than synthetic ones due to their compatibility, availability, easy processing, and tunable properties. A schematic outline of biopolymers for food packaging is given in Fig. 12.

#### 4.3.1. Natural biopolymers

##### 4.3.1.1 Proteins

Proteins are highly biocompatible and biodegradable components possessing multiple binding sites for the encapsulation of bioactive components.^159^ Even though protein-based electrospun nanofibers have several advantages, the electrospinning of proteins themselves is quite challenging due to their complex secondary and tertiary structure.^160^ Depending on the protein type, there are many other ways to make proteins favorable for electrospinning such as blending with other biopolymers, denaturation of protein structure, and use of appropriate solvent systems.^161^

**Fig. 13:**
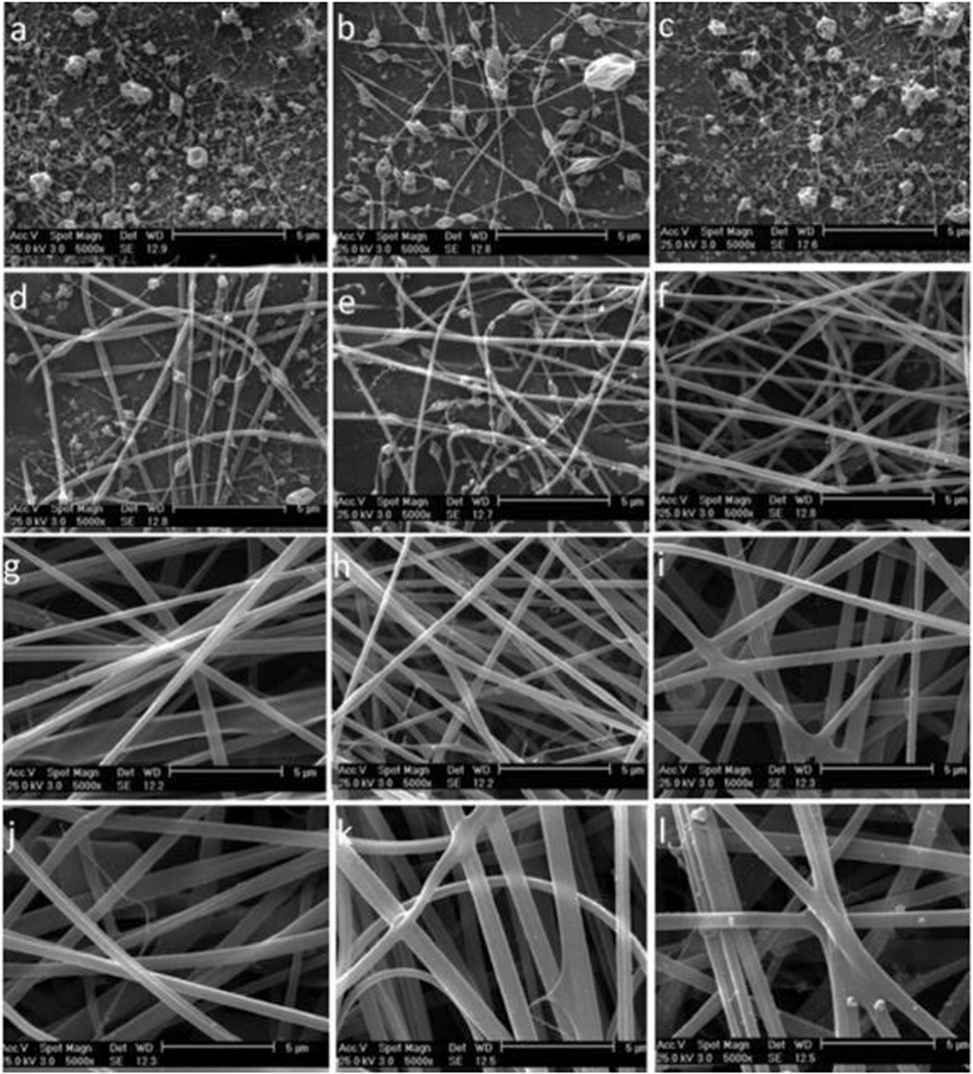
SEM images of WPI–GG electrospun nanofibers at varying whey protein isolate (WPI) and guar gum (GG) concentrations. Images (a–c), (d–f), (g–i), and (j–l) correspond to increasing WPI concentrations of 5%, 7%, 9%, and 12% (w/w), respectively, arranged as rows, each column represents different GG concentration (0.7%, 0.8%, and 0.9% w/w) at a resolution of 5 μm [reprinted with permission from ref. 162 Copyright 2019 Elsevier].

Whey protein isolate (WPI) is one of the main biopolymers used in food packaging materials due to its film-forming and gas barrier properties.^163,164^ WPI is an excellent carrier of both bioactive and pharmaceutical compounds, hence it has wide-ranging applications in the food industry and biomedical field. It is isolated from milk by-products and is added to improve the textural and nutritional values in food formulations due to its superior gelling and emulsifying properties. Since the electrospinning of pure whey protein is challenging, Mohammadi *et al.*^162^ evaluated the spinnability of whey protein isolate (WPI) at various concentrations with the addition of guar gum (GG), demonstrating that fiber formation is significantly influenced by the WPI-to-GG ratio. At a concentration of 7% WPI with 0.9% GG, fibers containing beads were observed, whereas no fibers formed at lower WPI concentrations (5%), irrespective of the GG content. This indicates that adequate chain entanglement, essential for electrospinning, occurs only at specific WPI/GG ratios. Higher WPI concentrations (9% and 12%) further enhanced spinnability, resulting in uniform, bead-free nanofibers with diameters of approximately 466 ± 87 nm and 510 ± 77 nm, respectively (Fig. 13). Panahi *et al.*^165^ fabricated PVA/WPI fibers encapsulated with probiotics as a novel platform for active food packaging to improve the food safety and to extend the shelf life. *Bifidobacterium bifidum* was successfully encapsulated within the optimized nanofiber system and these systems demonstrated effective antimicrobial properties against *L. monocytogenes* and *E. coli.* A color indicator film based on whey protein isolate nanofibers incorporated with anthocyanin as an indicator was developed by Han *et al.*^166^ This pH indicator film was used for sensing the pH of salmon, which changes with deterioration. Anthocyanin displayed a color shift from dark pink to grey with the increase in pH. Additionally, glycerol and pullulan were also incorporated with the film to improve its plasticizing and mechanical properties.

**Fig. 14:**
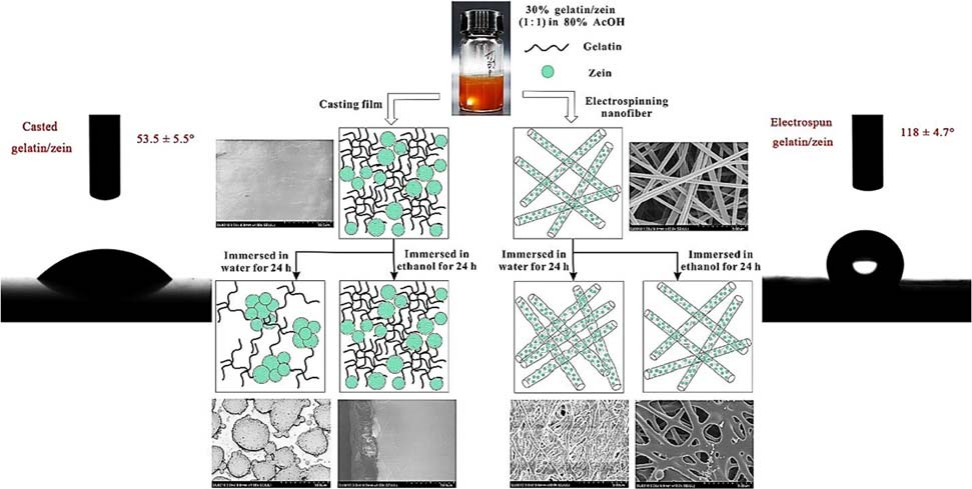
Morphology of the electropsun and cast zein/gelatin films after immersion in water and ethanol for studying solvent resistance capability and their water contact angle. [Reprinted with permission from ref. 167 Copyright Elsevier 2018].

Zein is another important protein known for its biodegradability, biocompatibility, hydrophobicity and film-forming properties. It is a prolamine protein obtained from maize corn.^168^ Zein-based systems are exclusively studied as food packaging and edible coating materials. Recently, bioactive compounds are incorporated into zein nanofibers for smart food packaging applications due to their resistance to moisture, oxidation, and heat.^169^ In a study conducted by Deng *et al.*, gelatin/zein electrospun membranes and solvent-cast films were fabricated to find out which one has the greater potential for food packaging applications.^167^Fig. 14 shows the morphology of electrospun and cast gelatin/zein films immersed in water and ethanol to study the solvent resistance. Gelatin/zein nanofibers showed good solvent resistance to water and ethanol, while cast films exhibited poor solvent resistance. Interestingly, the electrospun film had a hydrophobic surface with a water contact angle of 118° and the cast film had a hydrophilic surface with a water contact angle of 53°. The high water contact angle observed for the electrospun membranes is due to the formation of hydrogen bonding between zein and gelatin structures. Thus, the results confirmed the suitability of electrospun gelatin/zein nanofibers rather than the corresponding solvent-cast films for food packaging applications. Similarly, electrospun PVA/gelatin/zein membrane-based active food packaging developed by Ullah *et al.*^170^ and tetradecane-encapsulated zein nanofibers for sausage packaging^171^ are some other examples of zein-based nanofibers for smart food packaging applications.

Gelatin is one of the versatile biopolymers used for food packaging and it can be formed by the hydrolysis of collagen, the most abundant protein in animals.^172^ Gelatin is rarely used alone due to its limiting properties like brittleness, low bioactivities, and highly hydrophilic nature. Thus, they need to be modified by crosslinking, grafting, and blending to overcome its limitations in food packaging applications.^173^ Electrospinning of gelatin can be achieved in mild solvents such as a mixture of acetic acid/water and ethanol/formic acid/water. Yavari *et al.*^174^ fabricated a novel electrospun gelatin nanofiber reinforced using oxidized xanthan gum (OXG) as a crosslinking agent. The presence of OXG reduced water vapor permeability, water solubility, and moisture content, while their thermal and mechanical properties were changed.

Recently, a pH-responsive membrane based on gelatin and red anthocyanin extract was developed by Chayavanich *et al.*^175^ for the real-time monitoring of meat spoilage. The developed membrane exhibited a distinct colour change with the change in pH as a result of the volatile basic compounds released from spoiled meat. Similarly, Duan *et al.*^176^ fabricated a multifunctional food packaging system based on gelatin/chitosan nanofibers loaded with curcumin as a novel active and intelligent packaging material. The incorporation of curcumin has improved its antioxidant and antibacterial activities. Moreover, the developed electrospun membrane acts as a colorimetric indicator in ammonia sensing for monitoring the freshness of meat and seafood.

**Fig. 15:**
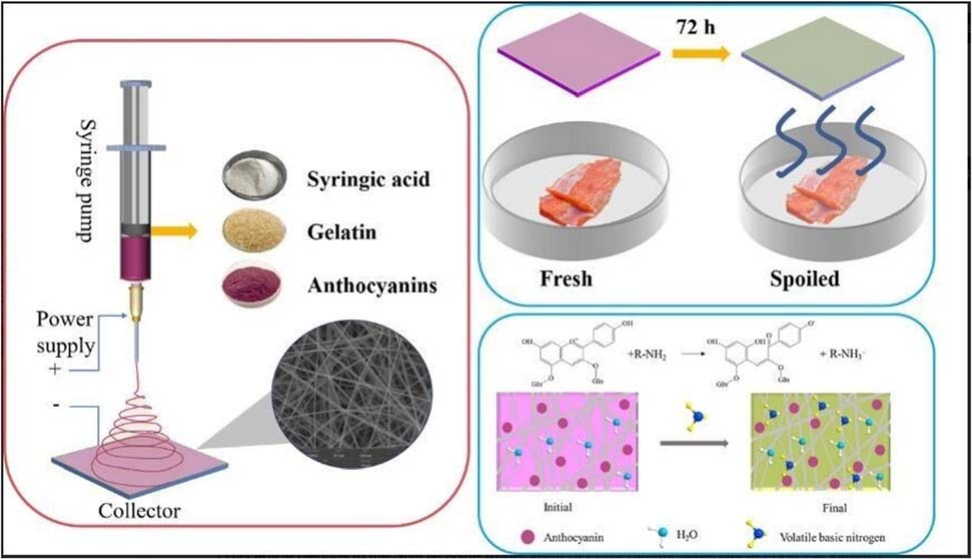
Schematic representation showing the fabrication and application of the GA/PPA/SA nanofiber membrane for pork preservation and freshness monitoring.^177^

Huang *et al.*^177^ developed an innovative electrospun gelatin nanofiber membrane incorporating purple potato anthocyanin (PPA) and syringic acid (SA) for the purpose of preserving and monitoring the freshness of pork (Fig. 15). Syringic acid, a plant-derived phenolic compound, is known for its significant antibacterial and antioxidant properties. These biological activities are primarily attributed to the presence of hydroxy (–OH) and methoxy (–OCH_3_) functional groups within the aromatic rings. The study thoroughly examined the impact of SA on mechanical strength, thermal stability, and antioxidant and antibacterial activities. An optimized SA loading was found to enhance the thermal stability and mechanical strength *via* intermolecular hydrogen bond interactions. Furthermore, GA/PPA/SA nanofibers demonstrated over 80% antioxidant activity and exhibited strong antibacterial efficacy against both *E. coli* and *S. aureus*. The inhibitory mechanism involves damage to the cell membrane, increased membrane permeability, and elevated intracellular pH. In pork spoilage tests, the ammonia sensitivity response resulted in a noticeable color change from purple to green. These findings indicate the suitability of the GA/PPA/SA nanofiber membrane for multifunctional food packaging applications.

Casein is a thermally stable phosphoprotein present in milk and cheese. Casein is responsible for the white color of milk. Due to their high nutritional value, water solubility, emulsification, non-toxicity, microbiological stability, and biodegradability, the milk proteins casein and whey alone or in combination can be utilized for making edible films.^178^ Compared to polysaccharide-based edible films/coatings, the three-dimensional complexed structure of milk proteins generates films with greater stability, excellent barrier properties, and longer durability.^179^ Even though casein films have great potential to be used in food packaging, the presence of hydrophilic residues and the moisture-sensitive nature lower their mechanical strength, thereby limiting their commercial acceptance. Crosslinkers and plasticizers are generally used to improve the protein film functionality.^180^ Sodium or calcium caseinates are the most commonly used caseinates for food packaging.

Pure sodium caseinate is not suitable for electrospinning due to the extensive network of inter- and intra-molecular hydrogen bonding. Therefore, suitable carrier polymers are required for the production of electrospun casein membranes. For example, polyethylene oxide (PEO) can be used as a suitable carrier polymer to make casein/PEO composites.^181^ The carrier polymer makes casein electrospinning feasible through the enhancement of solution viscosity and lowering of surface tension or electrical conductivity. Tomasula *et al.*^182^ have conducted a similar study to determine the feasibility of creating nanofibers from the food proteins calcium caseinate (CaCAS) and sodium caseinate (NaCAS) from an aqueous solution with and without a food-grade carrier polysaccharide, pullulan. They performed an extensive study to determine the effect of the solution and process parameters on fiber morphology. Similarly, Dai *et al.*^183^ fabricated a novel electrospun casein/PEO nanofiber membrane loaded with a thymol/β-cyclodextrin inclusion complex for beef preservation. Antibacterial studies revealed that casein nanofibers could facilitate the controlled release of thymol *via* the interaction of casein with bacterial proteases.

Soy protein isolate (SPI) is a plant-based protein originating from soybeans, and it is widely used in the food industry due to its biocompatibility, good film-forming nature, low cost, and high availability.^184^ The films made up of SPI will be transparent, smooth, and highly flexible. However, the main challenges associated with SPI films are their high water solubility and low mechanical stability. Additionally, electrospinning of pure SPI is also a challenge due to its low solubility in organic solvents and poor mechanical properties. These shortcomings can be rectified using suitable carrier polymers and functional components. PVA is an excellent carrier polymer that can provide required strength, and thus, it helps in the formation of nanofibers.^185^ Bruni *et. al.*^186^ developed an electrospun β-carotene-loaded SPI/PVA fiber mat as a bioactive coating for food packaging applications. Due to the lipophilic nature of β-carotene, emulsion electrospinning was used to encapsulate the carotenoid to a hydrophilic SPI/PVA hybrid system. Since proteins are excellent carriers for emulsion electrospinning, here SPI has been proposed as the carrier matrix for β-carotene. Therefore, SPI has been blended with spinnable biopolymer polyvinyl alcohol (PVA). A stable emulsion of SPI/PVA was prepared using soybean oil as the carrier oil for hydrophobic carotenoids. This emulsion was directly spun over a polyhydroxyalkanoate (PHA) film to form a continuous coating over it. They also studied the effect of annealing treatment on the controlled release of bioactive compounds from the electrospun membranes. The annealing process favored the adhesion of coating onto a PHA film and contributed to the sustained release of β-carotene. Similarly, Wang *et al.*^187^ fabricated functionalized electrospun SPI fiber mats using anthocyanin-rich raspberry extract as the active component. The film exhibited excellent antibacterial activity against *Staphylococcus epidermidis*, and hence, it could be used as an active packaging film. A novel bioactive electrospun nanofiber based on ethyl cellulose/SPI containing bitter orange peel extract was developed by Rashidi *et al.*^188^ Bitter orange peel, a rich source of phenolic compounds, makes this nanofiber membrane an excellent antioxidant and antibacterial active food packaging film.

##### 4.3.1.2 Polysaccharides

Polysaccharides are polymeric carbohydrates composed of long chains of monosaccharide units joined by glycosidic bonds with the general formula (C_6_H_10_O_5_)_*n*_, 40 ≤ *n* ≤ 3000.^189^ Polysaccharide-based packaging materials are promising solutions for reducing the dependence on petroleum-based plastics. Among biopolymers, polysaccharides are the most widely used raw materials since they originate from plant or marine organism's biomass. Due to their inherent characteristics such as biocompatibility, biodegradability and non-toxicity, polysaccharides are suitable for edible films and coatings in smart food packaging applications.^190,191^ Many polysaccharides (*e.g.* starch, cellulose, chitosan, alginate, carrageenan, dextran, and hyaluronic acid) have been used to obtain electrospun nanofibers for various intelligent applications. Protein-based nanofibers will undergo denaturation at elevated temperatures.^192^ Compared to protein-based nanofibers, polysaccharide-based nanofibers are most often used for high-temperature applications (nanoencapsulation) due to their excellent thermal stability. However, one of the major challenges with polysaccharide-based electrospinning is their low spinnability, which can be resolved by using various carrier polymers, co-solvents, and functional additives while preparing the electrospinning solution.^193^

**Fig. 16:**
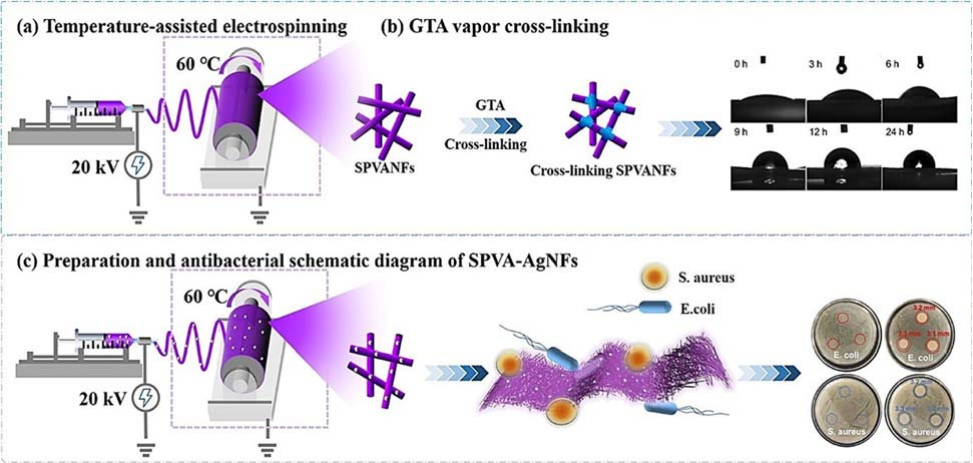
Schematic of (a) fabrication of SPVANFs, (b) crosslinking of SPVANFs with GTA vapors, and (c) preparation of SPVA-silver nanofibers (SPVA-AgNFs). [Reprinted with permission from ref. 199 Copyright Elsevier 2022].

Starch is a biodegradable natural polymer originating from rice, wheat, corn and potatoes. Starch mainly consists of two microstructures: linear structure known as amylose and branched structure known as amylopectin, whose properties and ratios vary depending on the source.^194^ Starch has great potential to form electrospun nanofibers due to its good biocompatibility, also it can be electrospun with other polymers such as polylactic acid (PLA), polyvinyl alcohol (PVA), polycaprolactone (PCL), polyepoxyethane and poly(ethylene-*co*-vinyl alcohol) to achieve combined features of these polymers.^195^ Starch electrospinning is quite challenging because of its semicrystalline nature, higher molecular weight, and extensive hydrogen bonding interactions.^196^ Heat annealing and cross-linking are some of the ways to enhance the crystallinity and wet stability of fibers. To improve the moisture sensitivity and mechanical properties of starch-based films, Zhu *et al.*^197^ developed crosslinked starch nanofiber (SNF) films using glutaraldehyde as the crosslinking agent. Electrospun SNF possessed higher flexibility and enhanced water sensitivity compared to solution-cast films. However, the presence of hydrophilic hydroxyl groups on the fiber surface and the disrupted crystalline region in starch contribute to the hydrophobic nature and poor mechanical strength to SNFs. A multifunctional soluble potato starch-carvacrol nanofiber was developed by Fonseca *et al.*^198^ with potential antimicrobial and antioxidant activities. Carvacrol is an excellent antioxidant and antimicrobial agent. It can accumulate on the cell membrane of bacteria, causing a 90% increase in the cellular permeability of microorganisms. This inhibits its growth and induces dehydration in bacterial cells. Liu *et al.*^199^ fabricated starch/PVA nanofiber composite films (SPVANFs) crosslinked with glutaraldehyde (GTA) and modified with silver sodium zirconium phosphate (Ag–ZrP) to improve their hydrophobic and antibacterial properties. The addition of glutaraldehyde improved the hydrophobicity of starch/PVA nanofiber films, and Ag–ZrP provided outstanding antibacterial properties against both Gram-positive and Gram-negative bacteria (Fig. 16).

Cellulose is one of the most abundant polysaccharides with unique properties. All natural fibers such as wood fibers, fruit, seed, and fruit fibers are examples of cellulosic fibers.^200^ Cellulose is resistant to strong alkalis but easily hydrolyzed by acids to soluble sugars. Due to the strong intra- and intermolecular hydrogen bonds and van der Waals interactions, it is insoluble in common organic solvents. The highly reactive and regularly arranged hydroxyl groups along the cellulose chains can be utilized to make a wide variety of cellulose derivatives *via* simple reactions between the functional substituents and active hydroxyl groups. Cellulose derivatives include various esters and ethers, such as cellulose acetate (CA), methylcellulose (MC), carboxymethyl cellulose (CMC), hydroxyethyl cellulose (HEC), and hydroxyl propyl methylcellulose (HPMC).

Since cellulose is not soluble in most common solvents, electrospinning is the best-known method to prepare cellulose nanofibers with versatile applications.^201^ Nowadays, cellulose nanofibers form the basis of functionalized materials and fiber-reinforced composites. Cellulose electrospinning is quite challenging due to its poor solubility; however, many research works have been published on the electrospinning of cellulose derivatives.^202,203^ In food packaging, cellulose and its derivatives are incorporated with active substances for spinning and the spun films act as carriers for active packaging. Baikzadeh *et al.*^204^ fabricated electrospun ethyl cellulose/polycaprolactone/gelatin (ECL/PCL/GEL) nanofibers incorporated with *Zataria multiflora* essential oil (ZEO) and zinc oxide (ZnO) nanoparticles for active food packaging applications. The ECL/PCL/GEL nanofiber with a weight ratio of 20/70/10, incorporated with 30% ZEO and 3% ZnO exhibited the highest biocompatibility, antioxidant, and antifungal properties. A biopolymeric pH indicator film based on cellulose nanofiber/chitosan dyed with methyl red (CCM) and coated with PLA was developed by Sobhan *et al.*^205^ and used for the real-time monitoring of beef and fish spoilage. The PLA/CCM films effectively biodegraded within 5 weeks and in response to pH variation and ammonia sensitivity test, the film showed noticeable color changes. In the study conducted by Pittarate *et al.*, an electrospun nanofiber film of cellulose acetate and polyethylene oxide (CA/PEO) blend was developed to study the effects of PEO on the morphology, moisture adsorption, and tensile properties of the film.^206^ The fibrous film fabricated by the electrospinning technique gives smooth, continuously long, ultrathin fibers with an average diameter of 800–900 nm. The addition of PEO significantly enhanced its mechanical properties and the incorporation of ZnO nanoparticles provided potential antibacterial properties to the film.

A multifunctional active and intelligent film based on cellulose nanofibers (CNFs) integrated with *Brassica oleracea* (BO) anthocyanin and BO-biowaste-derived carbon dots was fabricated by Wagh *et al.*^207^ The BO-carbon dot/CNF film is characterized by high UV-blocking capacity, and antibacterial and antioxidant properties compared to the neat CNF. BO anthocyanin enables the real-time freshness monitoring of pork, fish, and shrimp stored at 25 °C with a distinct color change from red to yellow. Corn straw core/CNF-based biodegradable nanocomposite film with bacteria blocking, UV, and water vapor barrier properties developed by Li *et al.*^208^ and CNF-based ethylene scavenging films incorporated with different types of TiO_2_ nanoparticles by Riahi *et al.*^209^ are examples of some recent works on cellulose nanofiber-based food packaging systems.

**Fig. 17:**
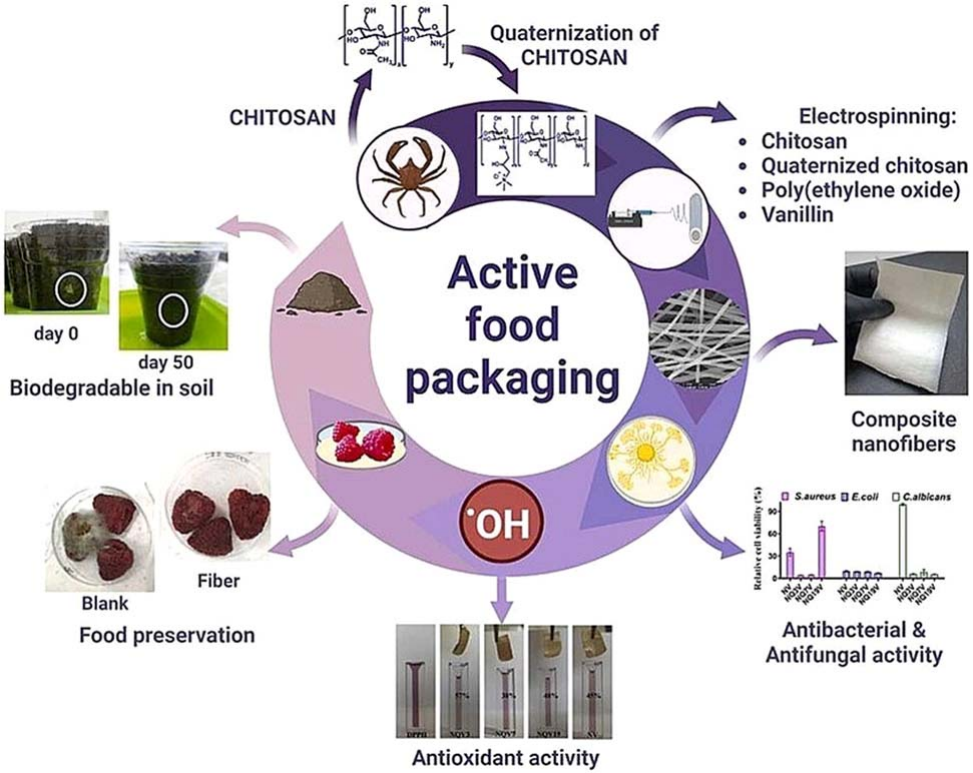
Quaternized chitosan/vanillin-based nanofiber membrane as an active food packaging material [reprinted with permission from ref. 214 Copyright 2023 Elsevier].

Chitosan is an amino polysaccharide obtained from the de-acetylation of chitin, the second most common biopolymer after cellulose.^210^ It is a copolymer of (1 → 4)-2-amino-2-deoxy-β-D-glucan and (1→4)-2-acetamido-2-deoxy-β-D-glycan. Chitosan is characterized by its low toxicity, high biocompatibility, biodegradability and good physical, chemical and biological (antibacterial) properties that promote their applications in food packaging, agriculture, cosmetics and biomedical field. Because of the highly hydrogen-bonded and semi-crystalline structure, it is difficult to dissolve both chitin and chitosan in most organic solvents. However, the solubility of chitosan can be enhanced through the functionalization of hydroxyl and amino-functional groups. Since chitosan is soluble in dilute acids, electrospinning solutions can be easily made in dilute acids such as acetic acid, trifluoroacetic acid (TFA), acrylic acid, and lactic acid.^211^ Recently, there have been many research works on electrospun chitosan nanofibers for antibacterial applications with the encapsulation of bioactive compounds such as essential oils, antioxidants, antimicrobial agents, and enzymes to develop functional materials for active food packaging.^212^ In a study conducted by Zou *et al.*^213^ electrospun chitosan/polycaprolactone nanofibers containing chlorogenic acid-loaded halloysite (CGA@HNT) were developed as active food packaging films. The CGA@HNT-loaded nanofiber mat exhibited long-term sustained release of bioactive components with enhanced antibacterial and antioxidant properties. Imino-chitosan/quaternized chitosan-based nanofibers with vanillin as antioxidant and antibacterial films for fruit preservation were developed by Andreica *et al.*^214^ The results revealed that the valuable combination of vanillin and quaternized chitosan into nanofibers proved to be effective in establishing a physical barrier against pathogens while maintaining the optimal humidity level. In the preservation of raspberries, the nanofiber membrane extended the shelf life to 7 days under normal atmospheric conditions (Fig. 17).

Similarly, several research studies have revealed the promising applications of chitosan-based electrospun nanofibers as active/intelligent food packaging to extend the shelf life and sensory properties of food products.^215–218^

Alginates are natural anionic polysaccharides found in the cell walls of certain brown seaweeds. The chemical structure of alginate consists of long chains of repeating units of two different monosaccharides, α-l glucuronic acid and β-d mannuronic acid.^219^ Alginates are extensively used for food and biomedical applications due to their biocompatibility, low toxicity, cost-effectiveness, and mild gelation by the addition of divalent cations such as Ca^2+^ and Ba^2+.^^220^ In food packaging applications, alginate-based films/coatings exhibit excellent film-forming properties, flexibility, water solubility, low water vapor permeability, and good tensile strength. When combined with various additives such as essential oils, plant extracts, metal nanoparticles, enzymes, and chelating agents, they offer a multitude of benefits. These include moisture retention, antioxidant, and antibacterial properties, preventing color and texture degradation, enhancing mechanical and barrier properties, and improving sensor acceptability.

Alginate being a naturally occurring polysaccharide tends to have a relatively high viscosity, which hinders the formation of smooth and continuous nanofibers during the electrospinning process. The electrospinnability of alginates can be improved through various approaches such as blending with synthetic polymers, ionic crosslinking, solution modification (viscosity, concentration), and fine-tuning the electrospinning parameters.^221^ Kyziol *et al.*^222^ demonstrated that the addition of a carrier polymer (PEO) and surfactant (Pluronic F-127) can generate uniform alginate nanofibers with a cylindrical shape. For the successful electrospinning of sodium alginate (SA) from aqueous solutions, Nie *et al.*^223^ introduced a strong polar co-solvent, namely glycerol. The results indicated that glycerol has improved the flexibility and entanglement of SA chains by disrupting the strong inter- and intramolecular hydrogen bonding among SA chains.

A novel electrospun nanofiber membrane based on zein/sodium alginate incorporated with TiO_2_ nanoparticles and betanin as an active food packaging system was developed by Amjadi *et al.*^224^ The combination of zein and sodium alginate helps in overcoming the drawbacks of pure zein (weak mechanical properties and rapid dissolution rate) and sodium alginate (high conductivity, surface tension, and low chain entanglements)-based nanofibers. Photocatalytic TiO_2_ nanoparticles with high antibacterial activity and betanin, rich in phenolic and cyclic amino groups, with high antioxidant properties loaded to zein/sodium alginate nanofibers are used as highly potential active food packaging systems for meat products, cheese, and cereals.

Pullulan is a natural polysaccharide consisting of repeating units of maltotriose residues linked by α-1,4-glycosidic bonds, with occasional branching through α-1,6-glycosidic bonds. It is produced from starch by the fungus *Aureobasidium pullulans*.^225^ Due to its unique properties such as excellent water solubility, biodegradability, biocompatibility, thermal stability, and non-toxicity pullulan finds applications in various industries including food, beverages, cosmetics, and packaging. Pullulan possesses exceptional characteristics such as remarkable flexibility and amorphous nature, enabling it to create a wide array of versatile products including thin layers, electrospun nanofibers, nanoparticles, flexible coatings and polymer films. These features make pullulan highly comparable to synthetic petroleum-derived polymers such as PVA and nylon 6, 6.^226^ Sun *et al.*^227^ fabricated pure pullulan nanofibers with a diameter of ∼100 to 700 nm using redistilled water as a solvent. They also studied the influence of various spinning parameters on the morphology and diameter of nanofibers. The optimized parameters were 22 wt% solution concentration, 31 kV voltage, 20 cm capillary-screen distance, and 0.5 mL h^−1^ flow rate.

**Fig. 18:**
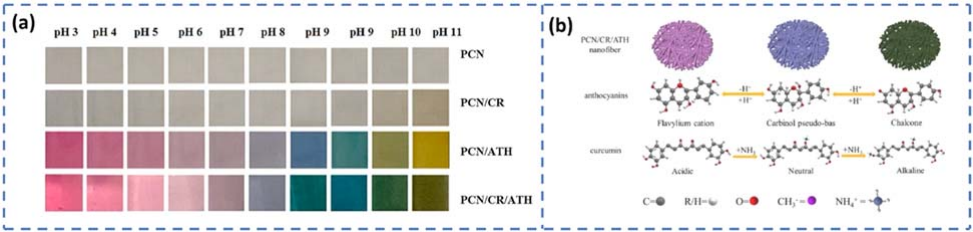
(a) Color changes in PCN/CR, PCN/ATH, and PCN/CR/ATH nanofiber membranes immersed in different buffer solutions (pH 2–11). (b) Mechanism behind the color change [reprinted with permission from ref. 230 Copyright 2021 Elsevier].

Recently, pullulan/polyvinyl alcohol nanofibers incorporated with a thymol-loaded porphyrin metal–organic framework have been developed by Min *et al.*^228^ as antibacterial food packaging materials. The developed nanofiber matrix system offered good flexibility, biocompatibility, and biodegradability. As an antibacterial packaging material, the nanofiber exhibited synergistic antibacterial activity against *E. coli* (∼99%) and *S. aureus* (∼98%) under light irradiation. Crosslinking is another way to enhance the properties of pullulan nanofibers because it reduces the hydrophilicity of nanofibers making them less susceptible to water absorption and it also reinforces the intermolecular interactions between pullulan chains, resulting in improved mechanical properties. Glutaraldehyde is the most commonly used crosslinking agent in biopolymer film making; however, some natural crosslinking agents are used these days to substitute the toxic agents. One such green crosslinking strategy was used by Quin *et al.*^229^ to fabricate electrospun chitosan/pullulan composite nanofiber films. In this study, cinnamaldehyde, the major component of cinnamon essential oil, was used as the green crosslinking agent. The crosslinked film showed superior water resistance, displaying hydrophobic characteristics along with enhanced mechanical and thermal properties. Electrospun pullulan/chitin nanofibers (PCN) loaded with curcumin and anthocyanin were developed by Duan *et al.*^230^ as an active-intelligent food packaging material. PCN containing both anthocyanin and curcumin (PCN/ATH/CR) had better antioxidant and antibacterial activities than nanofibers containing curcumin (PCN/CR) or anthocyanin (PCN/ATH) alone. In response to pH, PCN/CR did not show any significant color change, but PCN/ATH and PCN/ATH/CR fibers exhibited significant color changes (Fig. 18). An antibacterial food packaging system based on core–shell nanofibers with pullulan as the core layer and carboxymethyl chitosan/PEO as the shell layer loaded with nisin nanogels was developed by Duan *et al.*^231^ Nisin is a promising natural antibacterial agent against Gram-positive bacteria. Due to its high sensitivity to temperature and pH, coaxial electrospinning was chosen for the stabilization of nisin in food packaging materials. A tea polyphenol-loaded pullulan/carboxymethyl cellulose nanofiber membrane for fruit preservation developed by Shao *et al.*^232^ and an antioxidant electrospun nanofiber membrane based on pullulan/amaranth protein isolates loaded with curcumin by Blanco *et al.*^233^ are few examples for smart food packaging applications based on pullulan nanofibers.

Dextran is a bacterial polysaccharide synthesized by lactic acid bacteria or their enzymes in the presence of sucrose.^234^ Dextran is composed of an α-1,6-glycosidic linkage in which d-glucose units are linked by α-(1 → 2) or α-(1 → 3) or α-(1 → 4) bonds. The properties of dextran vary with their molecular weight and branching. The solubility of dextran in both water and organic solvents makes it a versatile biopolymer for preparing nanofiber membranes. They can be blended with water-soluble bioactive agents or hydrophobic biodegradable polymers to tailor the properties for food packaging applications. Luo *et al.*^235^ fabricated a curcumin-loaded dextran/zein hybrid nanofiber membrane as an antioxidant packaging film. A curcumin-encapsulated dextran/zein fiber membrane made from a solution comprising 50% dextran and 30% zein exhibited desired functional and mechanical properties. While there is limited research on electrospun dextran-based membranes for food packaging applications, film-based systems are more prevalent. Davidovic *et al.*^236^ developed a dextran-based edible film for the preservation of blueberries. The film was plasticized with different concentrations of polyglycerol. The results from the studies conducted on both coated and non-coated samples suggested the effectiveness of dextran/polyglycerol coatings to extend the shelf life of blueberries. A biodegradable antibacterial and antioxidant nanocomposite film based on Dextran/PVA/TiO_2_ developed by Islamipour *et al.* and an antioxidant trilayer film of gelatin/dextran-propyl gallate/gelatin by Wang *et al.*^237^ are examples of dextran-based systems for active food packaging.

#### 4.3.2. Synthetic biopolymers

Polyhydroxyalkanoates (PHAs) are natural polyesters produced by microorganisms under nutrient limitations (nitrogen, phosphorous or oxygen) for intracellular carbon and energy storage purposes.^238^

Polyhydroxybutyrate (PHB), polyhydroxyvalerate (PHV), and polyhydroxyhexanoate (PHH) are some of the most commonly used PHAs in biomedical and food packaging fields. Even though PHAs are biodegradable and biocompatible, their low mechanical strength, thermal properties, high production cost, and poor functionalities limit their applications in various fields. To overcome these limitations, they have to be modified to ensure better performance for specific applications. Blending with natural biopolymers such as starch, cellulose, and lignin is one way to improve their properties, and chemical modification *via* block and graft copolymerization is another way. However, electrospinning is gaining much popularity over other methods due to its remarkable versatility.^239^

Among various PHAs, the highly crystalline poly-3-hydroxybutyrate (P (3HB)) and its copolymers are the most extensively studied ones. Electrospinning helps to improve their mechanical properties and decrease the crystallinity. Aligned fibers offer better mechanical properties than randomly oriented fibers. Blended PHA fibers also render enhanced properties. Monolayered and multilayered electrospun PHA nanofiber membranes that can operate at room temperature can be synthesized *via* electrospinning by applying high electric voltages and annealing treatments.

Lopez *et al.*^240^ in their research study developed eugenol-incorporated ultrathin fibers of poly(3-hydroxybutyrate-*co*-3-hydroxyvalerate) (PHBV) and annealed them to obtain antimicrobial monolayers. It was found that 15 wt% eugenol was the optimal concentration in PHBV monolayers since it exhibited high electrospinnability and thermal stability as well as the most effective antibacterial reduction against *Staphylococcus aureus* (*S. aureus*) and *Escherichia coli* (*E. coli*). Several studies have reported on the application of bio-waste-derived electrospun PHBs incorporated with various essential oils and natural extracts as antioxidant and antimicrobial food package materials.^241,242^ Electrospun PHBV biopapers loaded with cerium oxide nanoparticles (CeO_2_ NPs) as oxygen-scavenging active food packaging films were developed by Lopez *et al.*^243^ The electrospun fibers were transformed into biopapers *via* an annealing process. Hexadecyltrimethylammonium bromide (CTAB) was used as the surfactant for loading CeO_2_ NPs into a PHBV solution. The best results for antibacterial, antioxidant, and oxygen scavenging performance were obtained for the PHBV film with 1.5 wt% CeO_2_ NPs and 5 wt% CTAB.

Polylactic acid (PLA) is a biodegradable, thermoplastic, renewable biopolymer obtained through the polymerization of lactic acid monomers. PLA can be synthesized by the bacterial fermentation of plant starch such as corn, cassava, potato, and sugar beet pulp. PLA possesses excellent characteristics such as high mechanical strength, biodegradability, biocompatibility, transparency, easy processability, and non-toxicity.^244^ It has been accepted as ‘Generally Recognized as Safe’ (GRAS) by the Food and Drug Administration (FDA) and hence suitable for direct food contact applications. Thus, PLA has been given special significance in food packaging applications as packaging films or coatings. Various processing techniques such as thermoforming and extrusion can be utilized to develop various PLA products such as cups, wrapping films, and food and beverage containers. However, electrospinning is one of the facile technologies for making PLA-based nanomaterials with enhanced gas barrier and thermal and mechanical properties. The nanofibers so obtained will have high porosity and large surface area-to-volume ratio, providing high encapsulation efficiency and controlled release of bioactive ingredients for active and intelligent food packaging applications.^245^

Wu *et al.*^246^ reviewed the recent advances and potential applications of PLA-based electrospun nanofibers for food packaging. By incorporating specific active agents or indicator substances, PLA fibers can act as antioxidant, antibacterial, or pH indicating fibrous mats that can provide information about food spoilage/contamination to the consumers. A novel antimicrobial electrospun nanofiber based on polylactic acid/hydroxypropyl methylcellulose (PLA/HPMC) containing pomegranate peel extract was developed by Bodbodak *et. al.*^247^ After optimizing various electrospinning parameters, they concluded that PLA/HPMC electrospun nanofibers in a concentration ratio of 80/20 with 10% pomegranate extract showed good fiber morphology with excellent antioxidant and antibacterial properties. Very recently, Liu *et al.*^248^ have developed a multifunctional PLA/butterfly pea flower extract (BPA)/cinnamaldehyde (CIN) (PLA/BPA/CIN) nanofiber film for monitoring the beef freshness. The nanofiber film exhibited remarkable antioxidant, antibacterial, hydrophobic, and colorimetric properties, which make it a potential intelligent packaging film for beef spoilage detection. Liao *et al.*^249^ modified electrospun PLA fibers with silver oxide-deposited hemp fibers as antibacterial fruit preservation films. Hemp fibers are natural fibers that can reinforce composite materials, and silver oxide has strong antimicrobial properties. Nanofiber membranes of the PLA/PEG blend incorporated with peppermint essential oil were produced *via* solution-blow-spinning (SBS). Peppermint essential oil (PO) is characterized by excellent antibacterial property due to the presence of l-menthol, menthone, methyl acetate, and limonene. SBS technique is a cost-effective technique, where a gas is used to accelerate the polymer solution into fiber. Thus, the PLA/PEG/PO film effectively extended the shelf life of strawberries.

**Fig. 19:**
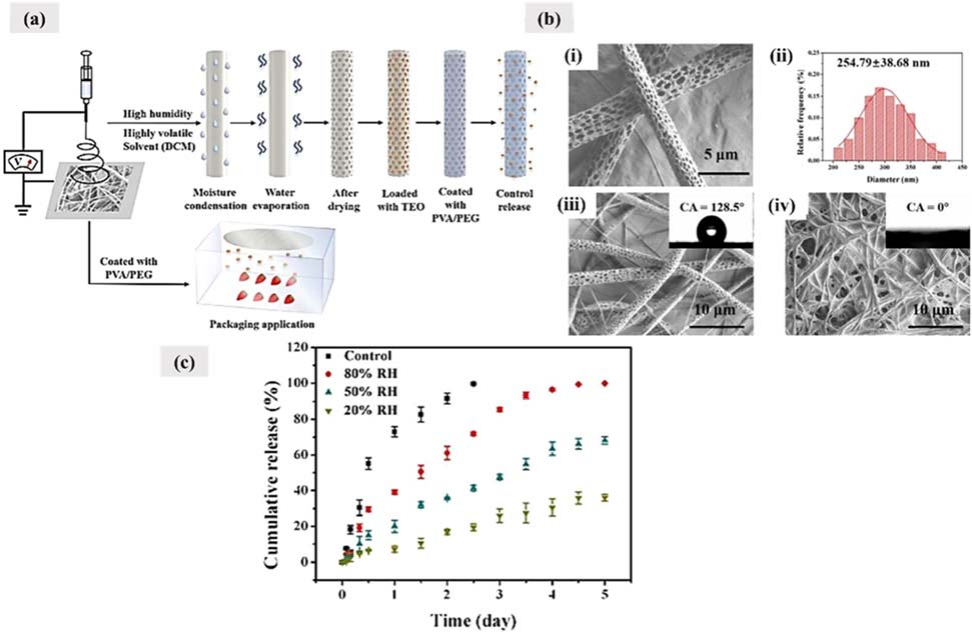
(a) Schematic showing the fabrication of PLA/TEO/PVA/PEG composite films using an electrospinning technique. (b) SEM images of (i) pure PLA nanofibers, (ii) pore size distribution, water contact angle of (iii) pure PLA nanofibers, and (iv) PVA/PEG coated PLA/TEO. (c) Cumulative release of TEO from the PLA/TEO/PVA/PEG composite film at different RH values. [Reprinted with permission from ref. 250 Copyright Elsevier 2020].

As a novel packaging solution, Min *et al.*^250^ fabricated an antimicrobial film based on porous PLA nanofibers with a hydrophilic PVA/PEG coating for the controlled release of thyme essential oil (TEO). A highly volatile solvent and a high relative humidity are the main factors that influence the formation of pores *via* phase separation mechanism as shown in Fig. 19a. TEO was successfully encapsulated within the pores of PLA nanofibers providing high loading capacity. However, it is still very difficult to achieve controlled release of antimicrobials from packaging films. Therefore, in this study, hydrophilic polymer molecules having swelling behavior that enhances their gas permeability when exposed to water vapor were used to develop a controlled release system. For that, PVA/PEG blend was coated onto a PLA/TEO fiber surface (PLA/TEO/PVA/PEG) and the *in vitro* release of TEO was controlled by adjusting the humidity (20% to 80% RH). The reduction in water contact angle from 128° to 0° confirms the hydrophilic nature of fibers with PVA/PEG coating (Fig. 19b). The release of TEO from the PLA/TEO/PVA/PEG composite film was very slow compared to PLA/TEO fibers. The cumulative release of TEO at different RH values is also given in Fig. 19c. The results indicated the increase in diffusion rate with the increase in relative humidity. This is because of the increased gas permeability facilitated by the swelling of polymer wall upon exposure to moisture. In addition to that, the composite exhibited excellent antibacterial action against *E. coli* and *S. aureus.*

## 5. Natural polyphenols as bioactive agents for smart food packaging applications

Polyphenols are plant-based naturally occurring bioactive compounds with excellent antioxidant and antibacterial properties. Based on the chemical structure, polyphenols can be classified into four main groups such as, phenolic acids, flavonoids, stilbenes, and lignans.^251^ Due to their inherent antioxidant and antibacterial properties, they can be used as functional components in active food packaging systems. The characteristic phenolic OH groups present in polyphenols help them to chelate with highly redox-active metal ions, which enhance their ability to protect against oxidative damage.^252^ Some commercial applications of polyphenols include functional food ingredients, antioxidants for preservation, and cosmetic ingredients. Raw fruits and vegetables are the main sources of polyphenols. Wheat, rice, maize, oats, coffee, tea, herbs, spices, and essential oils are the rich sources of phenolic compounds.^253^ Natural polyphenolic compounds such as curcumin, anthocyanins, tea polyphenols and gallic acid are widely used in biopolymer-based active food packaging films due to their diverse physiological, biological and beneficial health properties. Table 6 summarizes some recent research works related to natural polyphenols.

**Table 6:** Some recent research works related to natural polyphenols

Type of polyphenol	Source	Fiber matrix	Properties obtained	Ref.
Curcumin	*Curcuma longa L.* (turmeric)	Polycaprolactone-carboxymethyl chitosan (PCL-CMCH)	Encapsulation of curcumin enhanced thermal stability, tensile strength and hydrophobicity of PCL-CMCH nanofiber film	254
			Good biocompatibility, low toxicity and better inhibition of *S. aureus* under blue light irradiation	
		Pectin–gelatin	Composite film exhibited good antioxidant activity, mechanical properties and antibacterial activity against *E. coli* and *S. aureus*	255
			Excellent water vapor barrier capability	
			pH-responsive color change from yellow to dark red with increase in pH. Hence, suitable for monitoring the freshness of shrimp	
Anthocyanins	*Papaver rhoeas* extract	Guar gum–pectin	Color changes from white (fresh) to dark violet (spoiled) in the preservation of lamb meat	256
	*Black carrot extract*	PVA	Change in the morphology and structure of PVA-black carrot extract/SnO_2_ nanofiber film on interaction with released volatiles (confirmed by SEM, XRD, and FTIR) is due to the formation of complexes through new bonds	257
	Turnip peel extract	Potato starch	Turnip peel extract improved the thermal stability of potato starch nanofibers	258
			In the preservation of lamb meat, the nanofiber mat showed visible color change from white to purplish red in response to the microbial growth and chemical changes	
Tea polyphenols (TP)	Tea leaf extract	Starch	Tea polyphenol endowed antioxidant activity to the starch nanofiber film	259
			Hydrophobicity enhanced with addition of TP	
		High amylose corn starch and PVA	Addition of 15% TP enhanced the mechanical and water vapor barrier properties	260
			Effective antimicrobial activity against *S. aureus* by destroying the bacterial cell wall and degrading DNA fragments	
		Pullulan–carboxymethyl cellulose (CMC)	Decreased weight loss, maintained the firmness, and prolonged the shelf life of strawberries	261
		PVA-ethyl cellulose (EC)	Superior thermal stability of PVA-EC/-TP composite film compared to pure PVA nanofiber	262
			Significant increase in water contact angle from 22° to 109°	
			Reduced water vapor permeability	
			Good antioxidant and antibacterial properties	
Gallic acid		Pea flour-PEO	Thermal degradation of gallic acid decreased due to its successful encapsulation of gallic acid in the nanofiber matrix	263
			Total phenolic content of the film increased with gallic acid concentration	

Curcumin is one of the important polyphenolic natural bioactive compounds isolated from *Curcuma longa L.* (turmeric) belonging to the Zingiberaceae family.^264^ Among the three curcuminoid derivatives present in turmeric, curcumin (70%) is the principal curcuminoid. Curcumin has a yellowish-orange color with powerful antioxidant and antibacterial properties, which are attributed to the presence of *ortho-*methoxy and hydroxyl derivatives. Curcumin has many more biological functions such as anticancer, anti-inflammatory, and antimutagenic activities.^265^ Despite its reported benefits, one of the major problems that limits its bioavailability is poor water solubility, thermal sensitivity, and pH instability.^266^ Therefore, many studies have been conducted to overcome these limitations. One of the promising technologies is the encapsulation of bioactive agents in various delivery systems such as nanoparticles,^267^ nanoemulsions,^268^ nanofibers,^269^ β-cyclodextrin complexes,^270^ and biopolymer matrixes.^271^

**Fig. 20:**
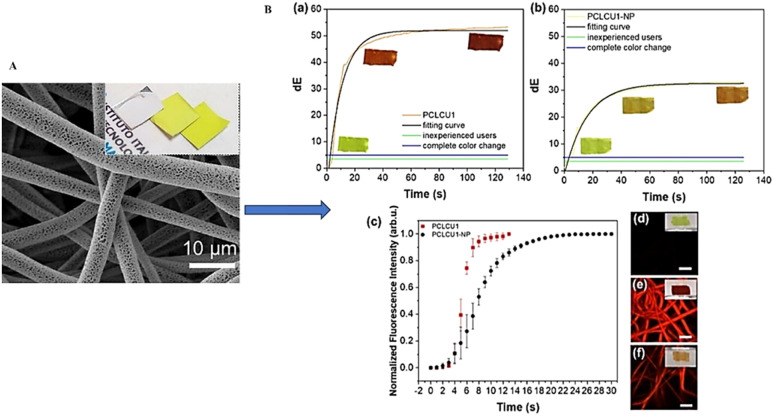
(A) SEM image of the porous PCLCU1 electrospun mat. (B) Kinetic curves showing CIELAB color space analysis, used to study the color responsiveness of the fibers upon exposure to dimethylamine (DMA) vapors: (a) response of PCLCU1 mat (b) response of nanoparticle-loaded PCLCU1 (PCLCU1-NP) mat (c) time taken by PCLCU1 and PCLCU1-NP mats to reach their maximum fluorescence intensity (∼640 nm) after DMA vapor exposure. (d) Photograph of the PCLCU1 mat before exposure to DMA vapors. (e) PCLCU1 and (f) PCLCU1-NP mats during exposure to DMA vapors, showing visible color change.^277^

In food packaging, curcumin-based systems are of great interest due to their good biocompatibility, low toxicity with food, UV protection, and free-radical scavenging properties.^272^ In intelligent food packaging, curcumin is used as a pH indicator with distinct pH-responsive color changes for the real-time freshness monitoring of food items.^273,274^ Recently, Du *et al.*^275^ have developed a green and efficient method for the delivery of active components to food by loading curcumin to a starch/pullulan nanofiber matrix. An inclusion complex of hydroxypropyl-β-cyclodextrin/curcumin was used to overcome the poor solubility of curcumin. The developed nanofiber membrane exhibited good antioxidant properties and fast dissolving since it was made in a 100% aqueous medium. A dynamically crosslinked chitosan/cellulose nanofiber integrated with a γ-cyclodextrin/curcumin inclusion complex was fabricated by Zhou *et al.*^276^ as a multifunctional packaging material for the preservation of perishable fruits. In this study, they used curcumin as a photodynamic antibacterial agent. Since curcumin is a natural photosensitizer, it generates reactive oxygen species (ROS) when exposed to blue light, resulting in bacterial cell death. In addition, the antioxidant property of curcumin helps to reduce the respiration of fruits and helps in prolonging their shelf life. A highly porous colorimetric indicator based on curcumin-loaded (1 wt%) polycaprolactone nanofibers (PCLCU1) was developed by a non-solvent-induced phase separation technique.^277^ These porous membranes interact with amine vapors, causing reversible color changes that help in the real-time monitoring of food spoilage. A comparative study was also conducted with non-porous PCL-curcumin (PCLCU-NP) mats. Compared to the non-porous fibers, the developed system showed an immediate response to amine vapors with distinct color change even at very low vapor concentrations (2.33 ppm). Fig. 20 shows the reversibility of color of mats upon exposure to dimethylamine (DMA) vapors of specific concentrations.

Among polyphenols, anthocyanins are an interesting class of water-soluble flavonoid pigments found in fruits, vegetables, flowers, and leaves.^91^ Anthocyanins are responsible for the red-to-blue color of flowers and fruits. Besides, they have excellent antioxidant properties. Chemically, anthocyanins are glycosylated forms of anthocyanidins, sugar-free pigments in plants. The rich sources of anthocyanins include blueberries, grapes, red cabbage, radishes, currants, pomegranates, and black carrots.^278^ They possess a wide variety of biological and functional properties such as antioxidant, anticancer, antimicrobial, antiobesity, detoxification and antidiabetic effects.^279^

**Fig. 21:**
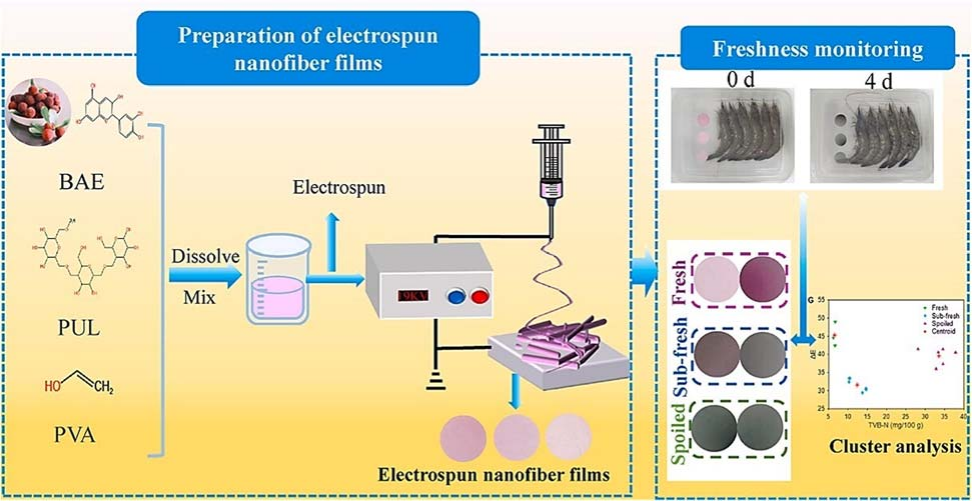
Pullulan/PVA nanofiber membranes incorporated with bayberry pomace anthocyanin extract for freshness monitoring. [Reprinted with permission from ref. 282 Copyright Elsevier 2024].

As a natural colorant, anthocyanins can be used as pH-responsive color indicators in smart food packaging applications like monitoring food quality and extending the shelf life of food products.^280^ Biopolymers such as polysaccharides and proteins are mainly used as solid supports for anthocyanins, since they are biodegradable, biocompatible, less-toxic, and eco-friendly. Recently, a starch-based electrospun nanofiber mat incorporated with roselle anthocyanin has been developed by Lv *et al.*^281^ for the real-time freshness monitoring of pork and shrimp. In this study, octenyl succinic anhydride starch (OSA) was used as the film forming material and PVA was added to enhance the electrospinnability of starch. During the spoilage of protein-based food items, the release of volatile base nitrogen increases, which causes an increase in the environmental pH level. Since roselle anthocyanin is a pH-sensitive dye, the fresh-to-spoilage transformation could be easily visualized as the color changed from red to dark green. Thus, this novel starch nanofiber mat-based smart food label can provide the best consumption period and spoilage information to the consumers. Jiang *et al.* fabricated^282^ electrospun pullulan/PVA nanofiber membranes with bayberry pomace anthocyanin extract (BAE) to monitor the freshness of aquatic products (Fig. 21). SEM, FTIR, XRD, and TGA results confirmed the successful loading of BAE to electrospun polymer matrixes. The pH value for fresh *Penaeus vannamei* is 6.92, which increases with deterioration and reaches a maximum value of 7.7. If the pH value exceeds 7.7 it is not good to consume. In this study, the shrimp freshness was monitored by dividing it into three states according to their TVB-N content: (i) ‘fresh’ state - TVB-N content <10 mg/100 g of shrimp, (ii) ‘sub-fresh’ state - 10 mg/100 g < TVB-N content < 30 mg/100 g, (iii) ‘spoiled’ state - TVB-N content ≥ 30 mg/100 g. The film efficiently differentiated the three freshness states (fresh, sub-fresh, and spoiled) of *Penaeus vannamei* during storage at 4 °C by exhibiting varying colors, from pink to grayish-purple to green.

Tea polyphenols (TPs) are the predominant bioactive agents present in tea leaf extract.^283^ TPs belong to a subclass of flavonoids, known as flavanols. Due to their effective antioxidant, antibacterial, anti-inflammatory, and preservative properties, TPs are widely used in various fields such as active food packaging, healthcare, and medicine.^284^ In active food packaging, TPs can act as excellent antioxidant and antibacterial agents. Moreover, through various interactive forces with the polymer matrix, they can improve the physical and chemical properties of the active film.^285^ Catechins, chemically flavan-3-ols, are the major components of TPs, which are primarily responsible for the antioxidant properties of green tea.^286^ The antioxidant efficacy of TPs is attributed to the presence of phenolic hydroxyl groups within polyphenol rings. The antioxidant mechanism of TPs involves its reaction with reactive oxygen species (ROS) to produce relatively stable phenolic oxygen radicals to scavenge free radicals.^287^ The antibacterial activity involves the attachment of TPs to the surface of the bacterial cell membrane through hydrogen bonding to produce hydrogen peroxide and damages the bacterial cell membrane by denaturing the proteins and disrupting the function of DNA and mRNA.

Liu *et al.*^288^ fabricated an antimicrobial PLA/TP nanofiber membrane for food packaging applications. The investigation on the release of TP from the PLA/TP composite fiber on food simulants such as 50% ethanol and 95% ethanol revealed the easy release of TP due to its excellent hydrophilic nature. For the initial 20 hours, there was a burst release followed by a slow and sustained release. This can be attributed to the fact that TP was adsorbed on the surface of PLA and randomly distributed over the PLA matrix by non-covalent interactions. The composite fiber exhibited the highest 1,1-diphenyl-2-picrylhydrazyl (DPPH) radical-scavenging capacity (95.07 ± 10.55%) and good antibacterial activity against *E. coli* and *S. aureus* (92.26% ± 5.93% and 94.58% ± 6.53%, respectively). A multifunctional active food packaging film based on gelatin nanofibers was developed by Lan *et al.*^289^ In this study, the antioxidant agent TP and the antibacterial agent ε-poly(l-lysine) were incorporated into a gelatin nanofiber membrane. The highest radical scavenging activity (>97%) and antibacterial activity (>93%) were obtained for 0.5% TP and 0.5% ε-poly(l-lysine) contents, respectively.

Gallic acid (GA) is a well-known natural antioxidant present in fruits, vegetables, and herbal medicines. They are secondary polyphenolic metabolites with the chemical formula C_7_H_6_O_5_ (3,4,5-trihydroxybenzoic acid).^290^ GA and its derivatives such as lauryl gallate, propyl gallate, octyl gallate, tetradecyl gallate and hexadecyl gallate have been found to possess antioxidant properties that inhibit oxidation by scavenging free radicals.^291^ Hence, they are useful additives in food preservation. In addition to the antioxidant effect, GA has many more biological functions such as antimicrobial, anticancer, and anti-inflammatory activities. Since GA is highly sensitive to temperature, pH, and light, it is usually encapsulated to conserve its bioactivity. Electrospinning is a versatile technique to encapsulate antioxidants susceptible to fast deterioration.

An effective antioxidant active food packaging material based on hydroxypropyl methylcellulose (HPMC) nanofibers was fabricated by incorporating GA to delay the oxidation of walnuts.^292^ Due to high amounts of polyunsaturated fatty acids, walnuts are highly prone to oxidation. HPMC nanofibers with 10% GA delayed the oxidation of walnut when compared to control. Moreover, the fiber diameter decreased with the increase in GA concentration. Chuysinuan *et al.*^293^ fabricated an electrospun PVA-based hydrogel composite with GA and studied their antioxidant activities by monitoring DPPH and ABTS radical scavenging assays and ferric reducing power. The highest activity was obtained for 7.5% GA/PVA electrospun fiber.

## 6. Sustainability and biodegradability of electrospun membranes

The sustainability of electrospun membranes largely depends on the choice of polymer. Biodegradable and bio-based polymers such as polylactic acid (PLA), polyvinyl alcohol (PVA), chitosan, gelatin, and cellulose derivatives degrade naturally in composting or normal soil conditions, compared to conventional petroleum-based plastics. These materials reduce both plastic waste and greenhouse gas emissions effectively.^294^ Replacing synthetic polymers with biodegradable polymers helps lower the carbon footprint while preserving the freshness of products. For example, PLA is fully biodegradable and industrially compostable biopolymer widely used for active food packaging applications. It is derived from renewable resources such as corn starch or sugarcane, making it an environmentally friendly alternative.^295^ Biodegradable electrospun membranes contribute significantly to sustainability by decomposing into harmless substances, thereby reducing long-term ecological effects. Recent advancements in the membrane technology field focus on green fabrication methods such as avoiding hazardous organic solvents, using renewable biopolymers, and facilitating membrane reuse or recovery.^296^ These advances align with sustainable packaging goals, offering eco-friendly alternatives to plastic materials.

While the recyclability of nanofiber membranes remains limited due to their complex morphology and contamination after use, their single-use environmental impact can be minimized by utilizing renewable materials and incorporating bioactive components. As regulatory and consumer demands are increasing progressively, the inclusion of green synthesis methods and biopolymer composites presents a viable approach towards sustainable, functional food packaging systems that align with circular economy goals.^158^ Even though electrospun membranes are biodegradable, their degradation rate is prolonged from six months to two years in a natural environment, highlighting the need for further research to accelerate biodegradation without compromising functional performance.

## 7. Conclusion and future outlooks

Advanced food packaging technology plays a pivotal role in ensuring food safety by preserving and extending the shelf life of food products without compromising their quality. Unlike traditional food packaging, active and intelligent packaging systems can convey the current quality status of food to consumers through visual color changes, written texts, and various other indicators. Although there are multiple methods to prevent microbial damage and contamination in food, such as incorporating preservatives directly into the food, adding toxic additives to extend shelf life, and employing UV/visible light irradiation, these approaches have certain limitations. The direct interaction of these substances with food can adversely affect its nutritional value and safety. Therefore, instead of using toxic preservatives, various bioactive agents are incorporated into packaging films, as they offer several advantages over direct addition to food. Biopolymer-based electrospun nanofibers have garnered significant attention as food packaging materials due to the growing environmental and health concerns associated with petroleum-based plastics. In comparison to films produced through alternative fabrication methods, electrospun nanofibers exhibit a high surface area-to-volume ratio, attributed to their small diameter and porous structure. These characteristics along with their compatibility with a diverse range of polymers, both natural and synthetic, facilitate the development of composite nanofibers with enhanced gas barrier, antimicrobial, and antioxidant properties.

Despite the numerous advantages discussed, the electrospinning technique also presents certain limitations. One of the major limitations of conventional electrospinning is its low productivity, hindering its large-scale industrial applications. Scaling up the process, while ensuring uniform quality and fiber characteristics, can be difficult. Safety concerns associated with electrospinning primarily come from the toxicity of the solvents employed in the process, as most biopolymers are water insoluble and hence require the use of organic solvents to dissolve them. The utilization of toxic organic solvents poses risks to human health and the environment, thereby reducing the environmental friendliness of this technology. Additionally, the incorporation of bioactive compounds to electrospinning solution poses further challenges, as many of these compounds are volatile and sensitive to external conditions. Exposure to high voltages during electrospinning may result in their degradation or inactivation, thereby compromising their functional performance. However, numerous electrospinning techniques have been developed to address these challenges. Coaxial electrospinning is a new technology that facilitates the encapsulation of sensitive compounds within a core–shell structure, thereby enabling controlled release and the utilization of un-spinnable materials. Multifluid electrospinning further enhances fiber complexity by incorporating multiple components. For large-scale production, multi-needle electrospinning increases output by enabling simultaneous spinning from multiple nozzles. Additionally, techniques such as emulsion electrospinning, blend electrospinning, needleless electrospinning, and near-field electrospinning offer improved control over the fiber structure and production efficiency, thereby advancing the applicability of electrospun materials in smart food packaging. Thus, by exploring innovative approaches, researchers and industries can develop advanced packaging materials that address consumers' demand and key challenges in food preservation, safety and sustainability.

## Author contributions

Maria Mathew: conceptualization and writing the original draft. Sagitha Paroly: review and editing. Sujith Athiyanathil: supervision, review, and editing.

## Conflicts of interest

There are no conflicts of interest to declare.

## Data Availability

No primary research results, software or code has been included and no new data were generated or analysed as part of this review.
